# A-FABP mediates adaptive thermogenesis by promoting intracellular activation of thyroid hormones in brown adipocytes

**DOI:** 10.1038/ncomms14147

**Published:** 2017-01-27

**Authors:** Lingling Shu, Ruby L. C. Hoo, Xiaoping Wu, Yong Pan, Ida P. C. Lee, Lai Yee Cheong, Stefan R Bornstein, Xianglu Rong, Jiao Guo, Aimin Xu

**Affiliations:** 1 State Key Laboratory of Pharmaceutical Biotechnology, LKS Faculty of Medicine, The University of Hong Kong, Hong Kong, China; 2 Department of Medicine, LKS Faculty of Medicine, The University of Hong Kong, Hong Kong, China; 3 Department of Pharmacology and Pharmacy, LKS Faculty of Medicine, The University of Hong Kong, Hong Kong, China; 4 Department of Medicine, University of Dresden, 01307 Dresden, Germany; 5 Joint Laboratory of Guangdong and Hong Kong on Metabolic Diseases, Guangdong Pharmaceutical University, 510000 Guangzhou, China

## Abstract

The adipokine adipocyte fatty acid-binding protein (A-FABP) has been implicated in obesity-related cardio-metabolic complications. Here we show that A-FABP increases thermogenesis by promoting the conversion of T4 to T3 in brown adipocytes. We find that A-FABP levels are increased in both white (WAT) and brown (BAT) adipose tissues and the bloodstream in response to thermogenic stimuli. A-FABP knockout mice have reduced thermogenesis and whole-body energy expenditure after cold stress or after feeding a high-fat diet, which can be reversed by infusion of recombinant A-FABP. Mechanistically, A-FABP induces the expression of type-II iodothyronine deiodinase in BAT via inhibition of the nuclear receptor liver X receptor α, thereby leading to the conversion of thyroid hormone from its inactive form T4 to active T3. The thermogenic responses to T4 are abrogated in A-FABP KO mice, but enhanced by A-FABP. Thus, A-FABP acts as a physiological stimulator of BAT-mediated adaptive thermogenesis.

## Abstract (web summary)

The protein A-FABP is secreted from adipocytes and known to regulate glucose and lipid metabolism. Here the authors show A-FABP enhances thermogenesis by promoting the conversion of thyroxine T4 to the bioactive form, T3, in brown adipocytes, and by enhancing fatty acid uptake of brown fat.

Obesity, which is caused by an extended energetic imbalance between energy intake and energy expenditure, is an important risk factor for type 2 diabetes and cardiovascular diseases^1^. Although the main function of white adipose tissue (WAT) is to store excess energy, brown adipose tissue (BAT), which is characterized by multi-locular oil vacuoles, high mitochondrial content and the presence of unique mitochondrial inner membrane protein uncoupling protein-1 (UCP-1), dissipates energy as heat^2^. Thus, BAT-mediated adaptive thermogenesis can be considered a defense mechanism that protects the organism against hypothermia or excessive weight gain in response to low temperature, or excess nutrient supply^3^. Accumulating evidence suggests that enhancing organismal thermogenesis is a promising therapy to combat obesity^4^.

Adaptive thermogenesis is primarily regulated by sympathetic nervous system (SNS), which heavily innervates interscapular BAT^3^. When the SNS is activated, catecholamines are released from sympathetic nerve endings and activate β-adrenoceptors on adipocytes, leading to an increase of intracellular cyclic adenosine monophosphate (cAMP) level. Elevated cAMP activates protein kinase A (PKA), which induces UCP-1 expression and activity^3^. Activated PKA also promotes lipolysis via stimulating hormone sensitive lipase (HSL) to provide free fatty acids (FFAs) as energy substrate for β-oxidation in mitochondria and UCP-1 expression^5^.

Thyroid hormones also contribute to adaptive thermogenesis in BAT by coordinating with SNS to induce expression of thermogenic genes^6^. Intracellular conversion of thyroxine (T4) into bioactive 3,3,5-triiodothyronine (T3) by type II iodothyronine deiodinase (D2) is required to activate the transcriptional program of thermogenic genes^7^. Other hormones, produced by various tissues, that have also been shown to induce BAT activity or browning of WAT include natriuretic peptides^8^, fibroblast growth factor 21 (ref. 9), irisin^10^, adipocyte-secreted leptin^11^, adiponectin^12^ and several type II immune cytokines (IL4, IL13 and IL33) (refs 13, 14, 15).

The adipokine adipocyte fatty acid-binding protein (A-FABP, also known as FABP4 or aP2) is abundantly expressed in adipocytes^16^, but also produced in macrophages^17^, endothelial cells^18^ and glial cells^19^. A-FABP functions as a lipid chaperone that regulates trafficking, fluxes and signalling of FFAs, and has an important role in linking lipid metabolism with inflammation^20^. Although A-FABP was originally identified as an abundant cytoplasmic protein in adipocytes, a portion of A-FABP is released into bloodstream and acts as a humoral factor to regulate glucose and lipid metabolism^21^,^22^. Circulating A-FABP is elevated in obese individuals and correlates positively with the features of the metabolic syndrome, and the incidence of atherosclerosis and cardiovascular diseases^18^. Interestingly, A-FABP knockout (KO) mice are protected against high-fat diet (HFD)-induced metabolic dysfunction but exhibit increased adiposity comparing with their wild-type (WT) littermates^23^. RNAi-mediated germline knockdown of A-FABP leading to a partial loss of A-FABP in mice also increases the susceptibility to diet-induced obesity^24^. Elevated A-FABP expression is observed in BAT of hibernating animals and cold-induced rodents^25^,^26^. Expression of *A-FABP* messenger RNA (mRNA) is also increased together with other thermogenic genes in BAT and WAT of HFD-induced UCP-1 deficient mice, suggesting that A-FABP might mediate a compensatory mechanism to maintain energy homeostasis^27^. A recent study demonstrated that ablation of both A-FABP and epidermal-FABP (E-FABP) impairs adaptive thermogenesis in mice in response to fasting and cold stress^28^. However, the underlying mechanism whereby A-FABP regulates energy metabolism remains elusive.

In this study, we found that the obese A-FABP KO mice have a marked attenuation of both HFD- and cold-induced BAT activation and energy expenditure, and this phenotype could be reversed by replenishment of recombinant A-FABP (rA-FABP). Mechanistically, we uncovered a role of A-FABP in promoting the intracellular conversion of T4 to T3 in BAT, and show that this is mediated in part by facilitating the transport of circulating FFAs released from WAT to BAT, which in turn enhances thermogenesis.

## Results

### A-FABP KO mice are defective in adaptive thermogenesis

**Figure 1:**
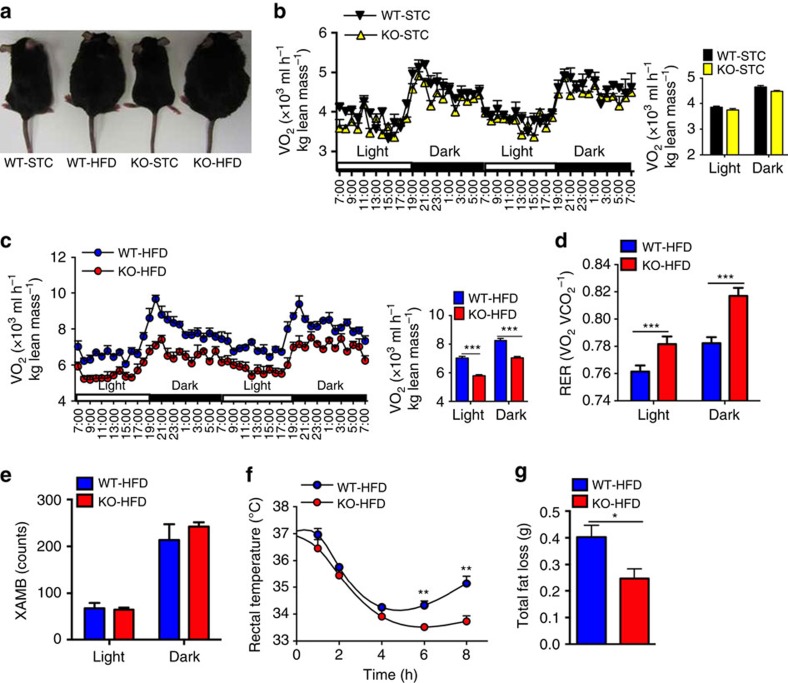
A-FABP deficiency impairs adaptive thermogenesis in mice. (**a**) Representative photos of male 4-week-old A-FABP KO mice and their WT littermates fed with either standard chow (STC) or high-fat diet (HFD) for 24 weeks (*n=*12). (**b**,**c**) Oxygen consumption (VO_2_) of the mice fed with (**b**) STC or (**c**) HFD for 4 weeks (*n=8*). (**d**) Respiratory exchange rate (RER) and (**e**) locomotory activity (XAMB) of above mice fed with HFD for 4 weeks (*n=*8). (**f**) Rectal temperature and (**g**) fat mass loss of A-FABP KO or WT mice fed with HFD for 24 weeks followed by cold exposure (6 °C) for 8 h (*n=*8). Data are represented as mean±s.e.m. *P<0.05, **P<0.01, ***P<0.001 (Student's *t*-test).

To explore the potential roles of A-FABP in the regulation of energy metabolism, global A-FABP knockout (KO) mice were generated^29^ and fed with standard chow (STC) or HFD. A-FABP KO mice are more susceptible to diet-induced obesity as compared with their WT littermates (Fig. 1a). After feeding with HFD for 24 weeks, body weight of WT mice was significantly increased by 43.3±2% and the body weight gain in A-FABP KO mice was even more drastic (∼106.7±2.5%) when compared with their respective STC-fed controls (Supplementary Fig. 1a). Body composition analysis showed no obvious difference in either lean mass or body fluid between A-FABP KO mice and WT controls on either STC or HFD. By contrast, the fat mass in HFD-fed A-FABP KO mice was 1.6-folds higher than the WT littermates (Supplementary Fig. 1b). This was further confirmed by dissection of mice showing a significant expansion in most of the fat pads in HFD-induced KO mice when compared with the respective WT controls (Supplementary Fig. 1c). No significant difference was observed in the weight of internal organs between WT or A-FABP KO mice. The weight of liver was even lighter in A-FABP KO mice (Supplementary Fig. 1d). Notably, there was no difference in the calorie intake between WT and A-FABP KO mice when fed with either STC or HFD (Supplementary Fig. 1e). Consistent with the previous study^23^, A-FABP KO mice exhibited an improved metabolic profile as indicated by the significant alleviation of HFD-induced glucose intolerance and insulin resistance (Supplementary Fig. 1f–h), a markedly reduced serum insulin and glucose levels, lipid profiles and a significantly higher adiponectin level compared with WT controls (Supplementary Fig. 1i–m).

Since A-FABP deficiency does not affect calorie intake, we next investigated whether HFD-induced morbid obesity in A-FABP KO mice was attributed to attenuated energy expenditure. The whole-body oxygen consumption (VO_2_) was comparable between STC-fed WT and A-FABP KO mice (Fig. 1b), while A-FABP KO mice fed with HFD for 4 weeks displayed a significantly lower oxygen consumption compared with the relative WT controls (Fig. 1c). HFD-induced A-FABP KO mice displayed a higher respiratory exchange ratio (Fig. 1d). In addition, glucose uptake in soleus muscle and liver was also significantly increased in A-FABP KO mice when compared with their WT littermates (Supplementary Fig. 1n), suggesting that A-FABP KO mice prefer to utilize carbohydrate rather than fatty acid as energy substrate. There was no obvious difference in locomotory activity (Fig. 1e) between HFD-induced WT and A-FABP KO mice.

To further verify the role of A-FABP in adaptive thermogenesis, HFD-fed WT and KO mice were housed at 6 °C for 8 h. Rectal temperature was dropped from ∼37 °C to 34 °C in the first 4 h in both the groups. Afterwards, the rectal temperature of A-FABP KO mice maintained at ∼34 °C while that of WT littermates gradually increased and significantly higher than that of A-FABP KO mice after prolonged cold exposure (Fig. 1f). Furthermore, A-FABP KO mice exhibited a significantly less fat loss compared with WT controls after cold exposure for 8 h (Fig. 1g). Taken together, these findings suggest that the morbidly obese phenotype of HFD-induced A-FABP KO mice is attributed to the impaired adaptive thermogenesis.

### A-FABP deficiency impairs BAT recruitment in mice

**Figure 2:**
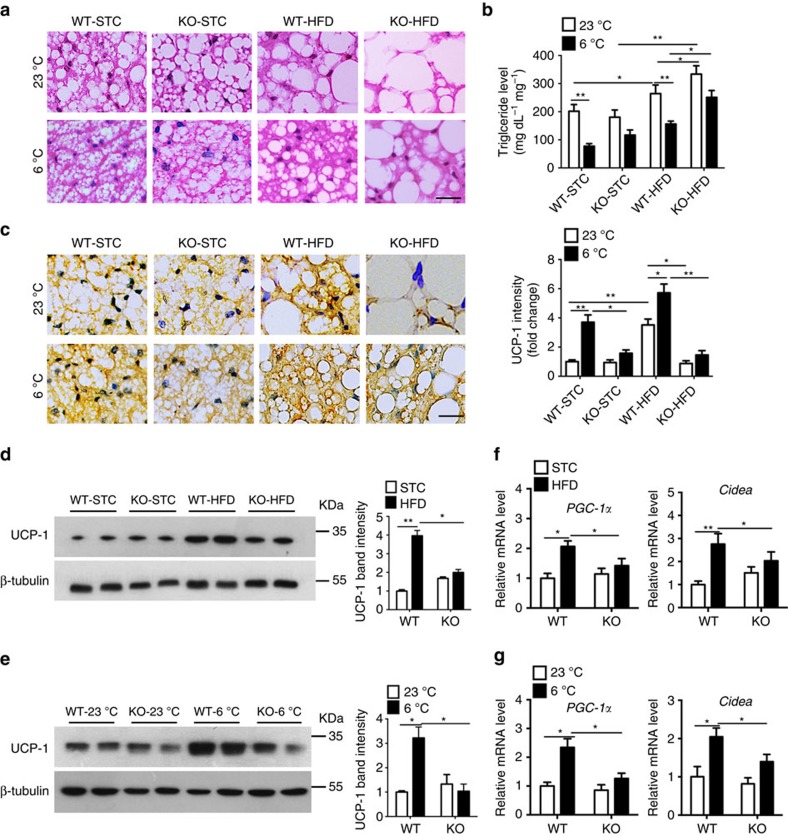
A-FABP deficiency impedes HFD- and cold-induced activation of BAT in mice. Male 4-week-old A-FABP KO and their WT littermates were fed with either STC or HFD for 24 weeks and subjected to room temperature (23 °C) or cold exposure (6 °C) for 24 h. (**a**) Haematoxylin and eosin (H&E) staining, (**b**) triglyceride levels, (**c**) immunohistochemistry (IHC) staining and densitometry analysis (right panel) for UCP-1 in brown adipose tissue (BAT) of mice. Scale bar, 20 μm, with magnification of 400 × . Representative images from three independent experiments are shown (*n=*8). (**d**,**e**) BAT isolated from above mice (**d**) fed with STC or HFD for 24 weeks or (**e**) exposed to 23 °C or 6 °C for 24 h were subjected to immunoblotting using an antibody against UCP-1, β-tubulin as indicated. Right panels are the band intensity of UCP-1 relative to β-tubulin and expressed as arbitrary units (*n=*8). (**f**,**g**) The mRNA abundance of the thermogenic genes in BAT of above mice (**f**) fed with STC or HFD for 24 weeks or (**g**) exposed to 23 °C or 6 °C for 24 h (*n=*8). Uncropped western blot images are shown in Supplementary Fig. 13. Data are represented as mean±s.e.m. *P<0.05, **P<0.01 (one-way analysis of variance with Bonferroni correction for multiple comparisons).

Brown adipose tissue (BAT) is the major organ responsible for adaptive thermogenesis. We next examined the impact of A-FABP deficiency in BAT recruitment in response to HFD and cold exposure. STC-fed A-FABP KO and WT mice showed comparable BAT morphology, while HFD-fed A-FABP KO mice displayed larger lipid droplets but reduced multi-locular structures comparing with WT controls (Fig. 2a, top). When exposed to 6 °C for 24 h, size and number of lipid droplets in HFD-fed WT mice were decreased more apparently than those in A-FABP KO mice (Fig. 2a, bottom). This was confirmed by measurement of triglyceride levels in BAT (Fig. 2b).

HFD or cold exposure induced approximately a fourfold increase of UCP-1 expression in BAT of WT mice, whereas A-FABP deficiency significantly attenuated cold- and HFD-induced expression of UCP-1 (Fig. 2c–e). Consistently, in response to HFD or cold challenge, A-FABP KO mice exhibited compromised induction in the expression of thermogenic genes (*PGC-1α* and *Cidea*) (Fig. 2f,g) while the expression of membrane fatty acid transporters such as CD36 and fatty acid transporter protein 1 were induced to a similar levels in both types of mice (Supplementary Fig. 2). Notably, A-FABP KO mice exhibited a similar cold-induced expression of UCP-1 and thermogenic genes in the subcutaneous fat as that in the WT littermates (Supplementary Fig. 3).

### A-FABP facilitates the transportation of FFAs into BAT

**Figure 3:**
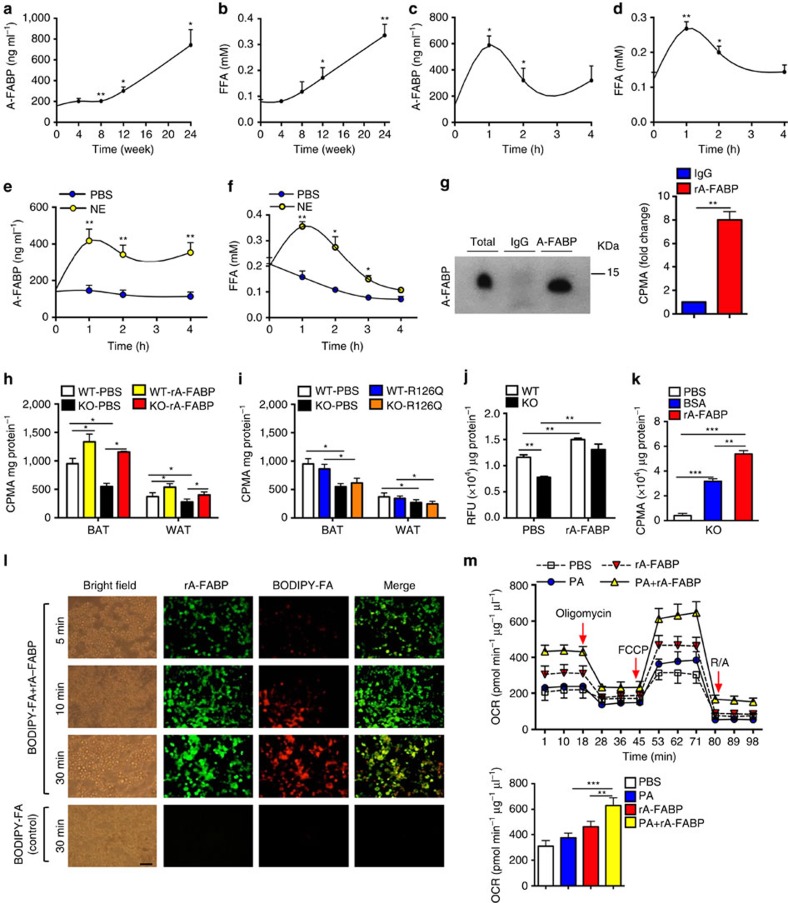
Circulating A-FABP facilitates the uptake of free fatty acid into adipocytes. (**a**) Circulating A-FABP and (**b**) FFA profile of male 4-week-old C57BL/6N mice fed with HFD for 24 weeks (*n=8*). (**c**) Circulating A-FABP and (**d**) FFA level of male 8-week-old C57BL/6N mice during cold exposure (6 °C) for 4 h (*n=*8). (**e**) Circulating A-FABP and (**f**) FFA level of male 8-week-old C57BL/6N mice intraperitoneally injected with norepinephrine (NE; 1 mg kg^−1^) or PBS (vehicle) for 4 h under fasting condition (*n=*6). (**g**) Co-immunoprecipitation (Co-IP) of A-FABP and ^3^H-palmitate in serum of male 8-week-old C57BL/6N mice after administration of ^3^H-palmitate (2 μCi) for 4 h. Right panel is the ^3^H-palmitate radioactivity of the co-immunoprecipitated A-FABP protein (*n=*6). (**h**,**i**) ^3^H-palmitate uptake in BAT and WAT of 8-week-old A-FABP KO mice and their WT littermates infused with PBS or (**h**) recombinant A-FABP (rA-FABP; 1 μg h^−1^) or (**i**) mutant R126Q (1 μg h^−1^) (*n=*6). (**j**) BODIPY-FA uptake in WT or A-FABP-deficient brown adipocytes treated with PBS or rA-FABP (2 μg ml^−1^) for 10 min (min) (*n=*6). (**k**) ^3^H-palmitate uptake in A-FABP-deficient adipocytes incubated with PBS, bovine serum albumin (BSA; 3 μg ml^−1^) or rA-FABP (2 μg ml^−1^) (*n=*6). (**l**) *In vitro* fluorescent imaging analysis of brown adipocytes treated with BODIPY-FA (2 μM) with or without pre-incubation with fluorescent-labelled rA-FABP (2 μg ml^−1^). Images were taken at 5, 10 and 30 min after treatment. Control image was taken at 30 min in which A-FABP-deficient brown adipocytes were incubated with BODIPY-FA without pre-incubation with rA-FABP. Scale bar, 20 μm, with magnification of 400 × . Representative images from three independent experiments are shown (*n=*6). (**m**) Oxygen consumption rate (OCR) and its mean value (lower panel) of A-FABP-deficient brown adipocytes treated with palmitate (PA: 200 nM) with or without pre-incubation with rA-FABP (2 μg ml^−1^) (*n=*6). CPMA, count per minutes for beta particles; RFU, relative fluorescence units; OCR, oxygen consumption rate; FCCP, carbonyl cyanide-4-(trifluoromethoxy) phenylhydrazone; R/A, rotenone/antimycin A. Uncropped image for co-immunoprecipitation is shown in Supplementary Fig. 13. Data are represented as mean±s.e.m. *P<0.05, **P<0.01, ***P<0.001 (Student's *t*-test (**a**–**g**), one-way analysis of variance with Bonferroni correction for multiple comparisons (**h**–**k**,**m**)).

A-FABP is not only a cytoplasmic protein, but also present in the circulation^21^. We next investigated the roles of circulating A-FABP in thermogenesis. Circulating A-FABP in C57BL/6N mice was progressively elevated upon feeding with HFD, and this change was accompanied by increased level of FFAs (Fig. 3a,b). When C57BL/6N mice were subjected to acute cold exposure, both circulating A-FABP and FFAs were increased to a peak level in 1 h, and gradually declined to a basal level (Fig. 3c,d). Similar results were observed in mice treated with the β-adrenergic receptor agonist norepinephrine (Fig. 3e,f), suggesting that circulating A-FABP may be released simultaneously with FFAs upon thermogenic stimulation. Notably, A-FABP abundance also increased markedly in WAT of C57BL/6N mice in response to HFD or cold exposure (Supplementary Fig. 4), suggesting that WAT may be the main source of elevated circulating A-FABP and FFAs. Therefore, we next tested whether A-FABP promotes adaptive thermogenesis by facilitating the transport of FFAs into BAT.

Infusion of ^3^H-palmitate in C57BL/6N mice followed by co-immunoprecipitation with anti-A-FABP antibody revealed that serum A-FABP could form complex with circulating FFAs (Fig. 3g). *In vivo* radioisotopic tracing showed that uptake of ^3^H-palmitate in BAT and WAT of A-FABP KO mice were significantly lower than that in their WT littermates (Fig. 3h) while replenishment with rA-FABP, but not its mutant R126Q which does not have binding capacity to FFAs^30^, significantly enhanced the uptake of ^3^H-palmitate in BAT and WAT (to a much lower extent) in both WT and A-FABP KO mice (Fig. 3h,i). This was further confirmed by the result of *in vivo* BODIPY-FA fluorescence chasing experiment (Supplementary Fig. 5a,b). Similarly, BODIPY-FA uptake was markedly attenuated in A-FABP-deficient primary brown adipocytes, while pre-incubation of BODIPY-FA with rA-FABP enhanced uptake of FFAs in both A-FABP deficient- and WT adipocytes (Fig. 3j). Furthermore, rA-FABP exhibited a higher efficiency than the classical FFA carrier bovine serum albumin (BSA) in promoting FFA uptake in A-FABP-deficient adipocytes (Fig. 3k). Fluorescent-labelled rA-FABP entered adipocytes together with BODIPY-FA simultaneously while no fluorescent signal was detected in the control in which no rA-FABP was added (Fig. 3l). Replenishment of rA-FABP but not its mutant R126Q significantly increased the FFA level in BAT and this change was accompanied by a decreased FFA level in WAT (Supplementary Fig. 5c) implicating that A-FABP acts as a FFA chaperone in transporting FFAs released from WAT to BAT. Notably, a significant portion of fluorescent-labelled rA-FABP administered through tail vein injection was delivered to BAT and WAT (to a lower extent). Cold exposure further enhanced the accumulation of rA-FABP in the BAT (Supplementary Fig. 5d,e). Furthermore, pre-incubation of palmitate with rA-FABP enhanced palmitate-induced oxygen consumption rate (OCR) in A-FABP-deficient adipocytes (Fig. 3m). Taken together, these data suggest that exogenous A-FABP facilitates the uptake of circulating FFAs into brown adipocytes to enhance its utilization.

### A-FABP enhances energy expenditure in A-FABP KO mice

**Figure 4:**
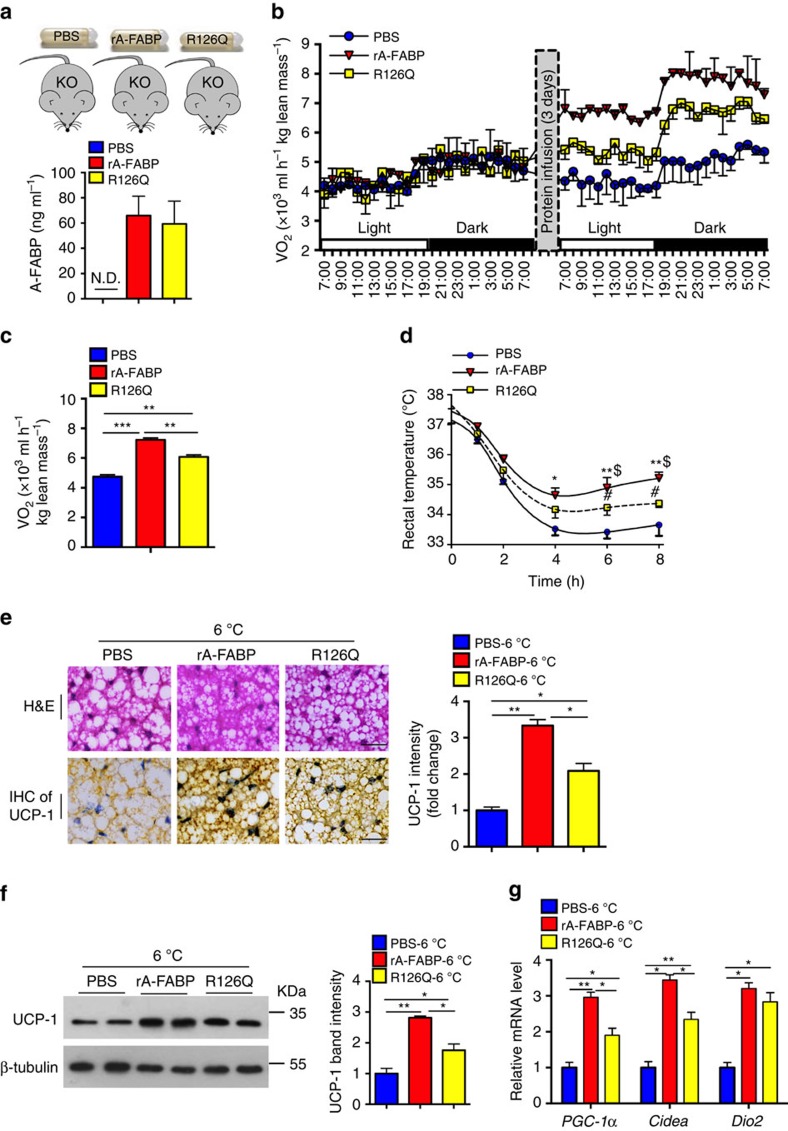
A-FABP enhances energy expenditure and BAT recruitment in A-FABP KO mice. Male 4-week-old A-FABP KO mice fed with HFD for 4 weeks were infused with PBS (vehicle), recombinant A-FABP (rA-FABP, 1 μg h^−1^) or A-FABP mutant R126Q (1 μg h^−1^) for 14 days with or without subjected to cold exposure (6 °C). (**a**) Circulating rA-FABP level and (**b**) oxygen consumption (VO_2_) of A-FABP KO mice before or after infusion of recombinant proteins (*n=*6). (**c**) Mean VO_2_ of above A-FABP KO mice measured after infusion of recombinant proteins for 3 days (*n=*6). (**d**) Rectal temperature of above A-FABP KO mice infused with rA-FABP or R126Q during cold exposure (6 °C) for 8 h. (**e**) Haematoxylin and eosin staining and IHC staining of UCP-1 in BAT of mice after cold exposure for 8 h, scale bar, 20 μm; with magnification of 400 × . The right panel is the densitometry analysis for UCP-1. Representative images from three independent experiments are shown (*n=*6). (**f**) BAT isolated from above mice was subjected to immunoblotting using an antibody against UCP-1, β-tubulin as indicated. The right panel is the band intensity of UCP-1 relative to β-tubulin (*n=*6). (**g**) The mRNA abundance of the thermogenic genes *PGC-1α, Cidea and Dio2* in BAT isolated from above mice (*n=*6). CPMA, count per minutes for beta particles. N.D., not detected. Uncropped western blot images are shown in Supplementary Fig. 13. Data are represented as mean±s.e.m. *P<0.05, **P<0.01, ***P<0.001*, $rA-FABP* versus *R126Q, $<*0.05*; #R126Q* versus PBS*, #P<*0.05 (One-way analysis of variance with Bonferroni correction for multiple comparisons).

To further determine whether circulating A-FABP promotes adaptive thermogenesis, rA-FABP or its mutant R126Q was continuously delivered into the circulation of 4-week HFD-fed A-FABP KO mice for a period of 2 weeks during which the whole-body energy expenditure was measured. rA-FABP and its mutant R126Q were detectable in a comparable level in the circulation (Fig. 4a), BAT and WAT (Supplementary Fig. 6a,b). The whole-body energy expenditure was significantly increased by 1.5-folds in rA-FABP-treated A-FABP KO mice compared with PBS-treated mice (Fig. 4b,c). The A-FABP KO mice infused with the mutant R126Q also showed an increase of oxygen consumption, but only to an approximately half extent as that of mice infused with rA-FABP (Fig. 4b,c). Notably, infusion of rA-FABP or R126Q caused a modest decrease in body weight and fat mass, whereas the calorie intake was not affected (Supplementary Fig. 6c,d).

To evaluate the effect of A-FABP in modulating BAT recruitment, A-FABP KO mice infused with the above proteins were subjected to 6 °C for 8 h. rA-FABP significantly increased the body temperature of A-FABP KO mice (Fig. 4d), suggesting an improved cold tolerance. However, this effect on cold tolerance was significantly attenuated in mutant R126Q-infused A-FABP KO mice (Fig. 4d). There was a remarkable increase of cold-induced multi-locular structures and upregulated UCP-1 expression in BAT of rA-FABP-infused KO mice (Fig. 4e,f), and were accompanied by a significantly elevated expression of thermogenic genes (*PGC-1 α*, *Cidea* and *Dio2*) (Fig. 4g) comparing to the PBS-infused control mice. However, the potency of the mutant R126Q in inducing the expression of UCP-1 and other thermogenic genes (except *Dio2*) were significantly lower than rA-FABP (Fig. 4f,g). Consistently, infusion with rA-FABP or R126Q to A-FABP KO and WT mice also modestly enhanced BAT recruitment at 23 °C but such an effect was much lower than their corresponding mice at 6 °C (Supplementary Fig. 6e–g and Supplementary Fig. 7). Notably, there was no obvious change in glucose tolerance, insulin levels and insulin sensitivity (as determined by the insulin resistance index) in these recombinant proteins-infused mice comparing to the PBS-infused mice (Supplementary Fig. 8a–c,f–h). The inflammatory status in the peripheral tissues was not altered in these mice (Supplementary Fig. 8d,e,i,j). Taken together, these data suggest that the fatty acid binding capacity of A-FABP is at least partially contributed to its ability for the enhancement of thermogenesis. However, the mutant R126Q was unable to increase FFA uptake while it could still partially reverse the impairment of adaptive thermogenesis in A-FABP KO mice, indicating that there may be an additional mechanism contributing to the ability of A-FABP in promoting thermogenesis.

### A-FABP deficiency impairs conversion of T4 to T3 in BAT

To explore additional mechanism whereby A-FABP regulates adaptive thermogenesis, we investigated whether A-FABP deficiency altered SNS activity. Oxygen consumption of A-FABP KO and WT mice on STC or HFD were comparable in response to norepinephrine (Supplementary Fig. 9a,b). Likewise, norepinephrine-induced circulating FFA levels were similar between HFD-induced WT and A-FABP KO mice (Supplementary Fig. 9c). HFD-induced expression of β adrenergic receptor 3 (*ADRB3*) and the activation of tyrosine hydroxlase in BAT were significantly increased to a similar level in both types of mice (Supplementary Fig. 9d,e). These data suggest that A-FABP deficiency does not attenuate SNS activity and lipolytic machinery in mice.

**Figure 5:**
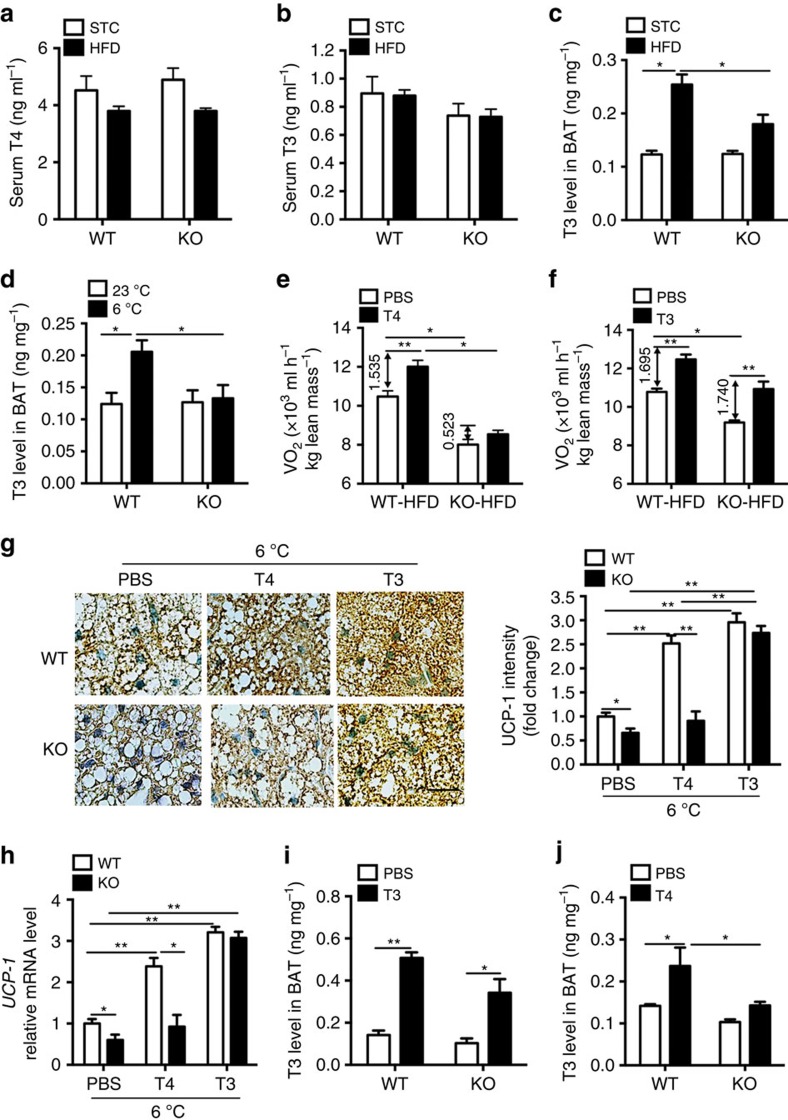
A-FABP deficiency impairs conversion of T4 to T3 in BAT of mice. (**a**) Circulating T4 and (**b**) T3 levels of male 4-week-old A-FABP KO mice and WT littermates fed with STC or HFD for 24 weeks as indicated in Fig. 2 (*n*=8). (**c**,**d**) T3 levels in BAT of male 4-week-old A-FABP KO mice and WT littermates (**c**) fed with STC or HFD 24 weeks or (**d**) subjected to cold exposure (6 °C) for 24 h as indicated in Fig. 2 (*n*=8). Male 4-week-old A-FABP KO and WT mice fed with HFD for 4 weeks were supplemented with PBS, T4 (400 μg kg^−1^, 5 days) or T3 (500 μg kg^−1^, 1 day) followed by cold exposure (6 °C) for 24 h. (**e**,**f**) Energy expenditure of mice supplemented with (**e**) T4 or (**f**) T3 followed by cold exposure (6 °C) for 24 h (*n=*6). (**g**) Representative IHC staining and densitometry analysis (right panel) for UCP-1 in the BAT of mice. Scale bar, 20 μM, with magnification of 400 × . Representative images from three independent experiments are shown (*n=*6). (**h**) The mRNA abundance of *UCP-1* in BAT of above mice (*n=*6). (**i**,**j**) T3 levels in BAT isolated from above WT and A-FABP KO mice fed with HFD for 4 weeks supplemented with (**i**) T3 or (**j**) T4 followed by cold exposure (6 °C) for 24 h (*n=*6). Data are represented as mean±s.e.m. *P<0.05, **P<0.01 (one-way analysis of variance with Bonferroni correction for multiple comparisons).

Since SNS and thyroid hormones regulate adaptive thermogenesis cooperatively^3^, we evaluated whether A-FABP deficiency impedes the activation of thyroid hormones. There was no difference in circulating T4 or T3 levels between A-FABP KO mice and their WT littermates fed with either STC or HFD (Fig. 5a,b). T4 is the major form of thyroid hormone in blood while it has to be converted to the activated form T3 within its target tissues. T3 levels in BAT of WT mice were increased significantly upon HFD feeding or cold exposure while this induction was abrogated in A-FABP deficient BAT (Fig. 5c,d). To investigate the effect of A-FABP in intracellular conversion of thyroid hormones, HFD-induced A-FABP KO and WT mice were supplemented with PBS or T4 for 5 consecutive days followed by cold exposure for 24 h. Supplementation of T4 enhanced cold-induced energy expenditure in WT mice, whereas such an effect of T4 administration was significantly attenuated in A-FABP KO mice (Fig. 5e). However, energy expenditure induced by supplementation of T3 was comparable between A-FABP KO and WT mice (Fig. 5f), implicating that A-FABP deficiency abolished T4 to T3 conversion in BAT. More multi-locular structures and elevated UCP-1 expression were observed in BAT of WT mice treated with either T4 or T3 (Fig. 5g,h). However, treatment with T3, but not T4, induced such changes in A-FABP KO mice (Fig. 5g,h). Similar results were observed in T4- or T3-treated WT and A-FABP KO mice under 23 °C (Supplementary Fig. 10a–d), although the magnitude of these changes was much smaller compared with those at 6 °C. The body weight, body composition and calorie intake were not altered in both types of mice under these circumstances (Supplementary Fig. 10e–h). Consistently, systemic supplementation with T3 markedly increased T3 levels in BAT of both genotypes (Fig. 5i), while treatment with T4 could only increase T3 levels in BAT of WT mice but not in A-FABP KO mice (Fig. 5j), suggesting that A-FABP is required for conversion of T4 to T3 within BAT.

### A-FABP enhances T3 levels in BAT via LXRα-*Dio2* pathway

**Figure 6:**
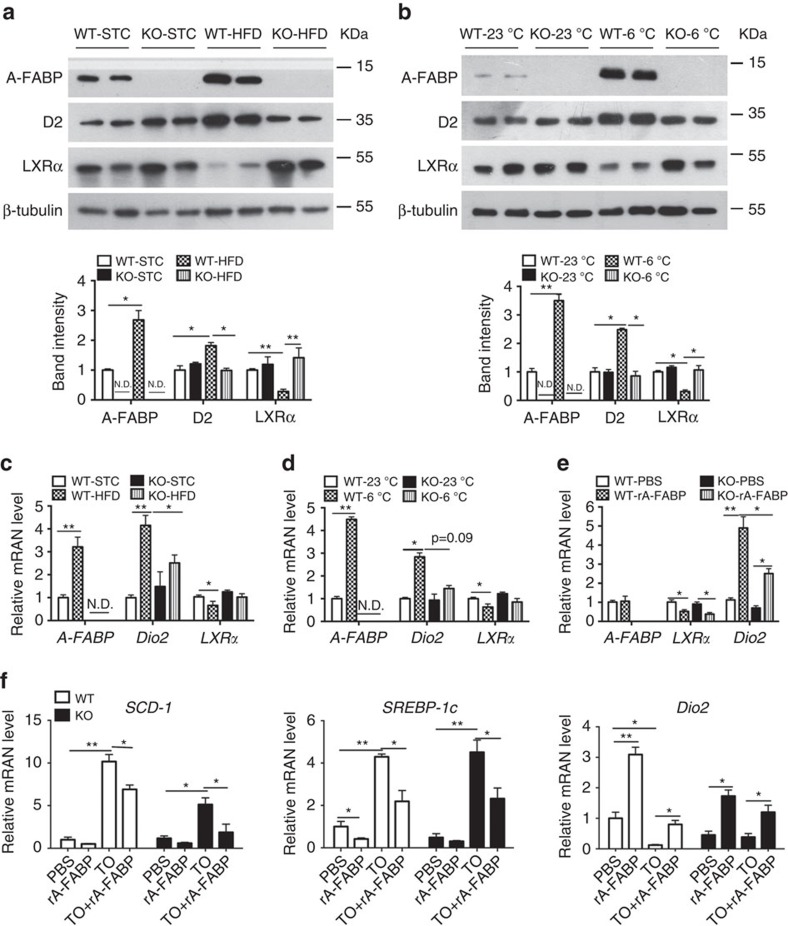
A-FABP mediates expression of *Dio2* via inhibition of LXRα. (**a**,**b**) BAT isolated from WT and A-FABP KO mice (**a**) fed with STC or HFD for 24 weeks or (**b**) subjected to room temperature (23 °C) or cold exposure (6 °C) for 24 h as indicated in Fig. 2 were subjected to immunoblotting using an antibody against A-FABP, type II iodothyronine deiodinase (D2), liver X receptor α (LXRα) and β-tubulin. The bar charts below are the band intensity of each protein relative to β-tubulin and expressed as arbitrary units, N.D., not detected (*n=6*). (**c**,**d**) The mRNA abundance of *A-FABP, Dio2* and *LXRα* in BAT of above WT and A-FABP KO mice (**c**) fed with STC or HFD for 24 weeks or (**d**) subjected to cold exposure (6 °C) for 24 h (*n=*6). (**e**) The mRNA abundance of *A-FABP*, *LXRα* and *Dio2* in WT or A-FABP-deficient primary brown adipocytes incubated with PBS or recombinant A-FABP (rA-FABP, 2 μg ml^−1^) for 24 h (*n=*6). (**f**) The mRNA abundance of LXRα downstream target genes (*SCD-1, SREBP-1c*) and *Dio2* in WT and A-FABP-deficient primary brown adipocytes treated with or without LXRα agonist TO901317 (TO;1 μM) and/or rA-FABP (2 μg ml^−1^) for 24 h (*n=*6). Uncropped western blot images are shown in Supplementary Fig. 13. Data are represented as mean±s.e.m. *P<0.05, **P<0.01 (One-way analysis of variance with Bonferroni correction for multiple comparisons).

Type II iodothyronine deiodinase (D2), which is encoded by the *Dio2* gene, is the key enzyme responsible for local T4 to T3 conversion^7^,^31^. *Dio2* is downregulated by the nuclear receptor LXRα^32^, while A-FABP represses LXRα activity and expression in macrophages^33^. Therefore, we investigated whether A-FABP regulates the conversion of T4 to T3 via LXRα-*Dio2* signalling pathway. HFD and cold exposure greatly induced the expression of A-FABP and *Dio2* in the BAT of WT mice, whereas this induction was obviously impaired in A-FABP KO mice (Fig. 6a–d). On the contrary, LXRα expression decreased substantially in WT mice in response to HFD or cold exposure, but its expression in A-FABP KO mice was not altered (Fig. 6a–d). Furthermore, treatment with rA-FABP did not alter the gene expression of *A-FABP* while it significantly suppressed *LXRα* but increased *Dio2* expression in both primary A-FABP-deficient and WT brown adipocytes (Fig. 6e). Treatment with the mutant R126Q also showed similar effects on the gene expression in both A-FABP deficient- and WT brown adipocytes (Supplementary Fig. 11), but to a lesser extent compared with those treated with rA-FABP.

To assess whether A-FABP represses LXRα activity, WT- or A-FABP deficient primary brown adipocytes were treated with the LXRα agonist TO901317 in the presence or absence of rA-FABP followed by monitoring the expression of the downstream target genes of LXRα, including stearoyl-CoA desaturase (*SCD-1*) and sterol regulatory element-binding transcription factor 1 (*SREBP-1c*)^34^,^35^. Treatment with TO901317 drastically increased the expression of *SCD-1* and *SREBP-1*c in primary brown adipocytes derived from both genotypes, which were greatly suppressed by co-treatment with rA-FABP (Fig. 6f). Consistently, the downregulated expression of *Dio2* by treatment with TO901317 was significantly reversed by rA-FABP (Fig. 6f), suggesting that A-FABP increases *Dio2* expression via inhibition of *LXRα.*

### A-FABP promotes proteasomal degradation of LXRα

**Figure 7:**
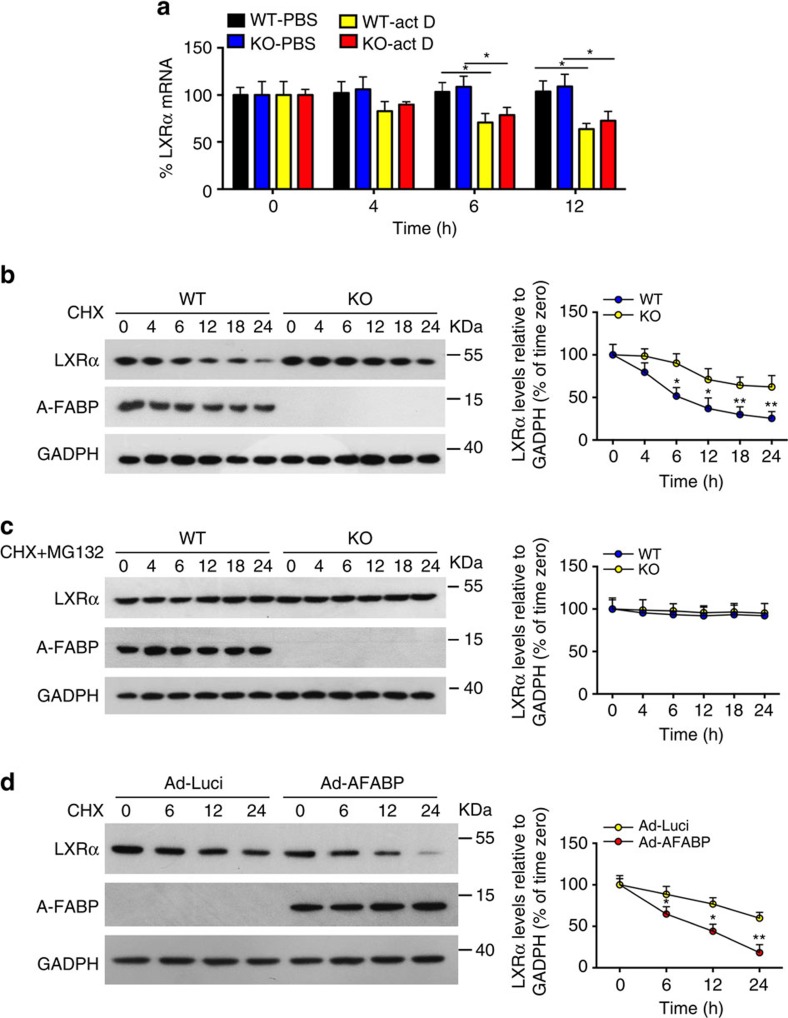
A-FABP accelerates proteasomal degradation of LXRα. (**a**) Primary adipocytes derived from male 6-week-old A-FABP KO mice or WT littermates were treated with actinomycin D (actD, 1 μg ml^−1^) or vehicle (PBS). The mRNA level of *LXRα* was determined by real-time PCR at time points as indicated (*n=*4). (**b**,**c**) Primary brown adipocytes derived from male 6-week-old A-FABP KO mice or WT littermates were treated with (**b**) cycloheximide (CHX, 50 μg ml^−1^) or (**c**) CHX (50 μg ml^−1^) together with MG132 (10 μM) for different periods were subjected to immunoblotting using an antibody against LXRα, A-FABP and GADPH as indicated (*n=*4). (**d**) Primary brown adipocytes derived male 6-week-old A-FABP KO mice or WT littermates were infected with adenovirus overexpressing A-FABP (Ad-AFABP) or luciferase (Ad-Luci) for 48 h, followed by treatment with CHX (50 μg ml^−1^) for 0,6,12 and 24 h, and then subjected to immunoblotting using an antibody against LXRα, A-FABP and GADPH as indicated (*n=*4). The right panel is the band intensity of LXRα normalized with GAPDH, and expressed as percentage relative to baseline (0 h). Uncropped western blot images are shown in Supplementary Fig. 14. Data are represented as mean±s.e.m. *P<0.05, **P<0.01 (Students' *t*-test).

To further explore the potential mechanism whereby A-FABP inhibits LXRα, A-FABP deficient- and WT primary adipocytes were treated with the transcription inhibitor actinomycin D followed by measuring the mRNA abundance of *LXRα* at different time points. A-FABP deficiency did not have significant effect on the basal mRNA level of *LXRα.* Furthermore, the mRNA abundance of *LXRα* was gradually decreased to a similar extent in both WT and A-FABP deficient adipocytes after treatment with actinomycin D (Fig. 7a), suggesting that A-FABP does not alter the mRNA stability of *LXRα.* We then examined if A-FABP modulates the protein stability of LXRα using cycloheximide (a protein synthesis inhibitor) chase assay. The degradation of LXRα in A-FABP deficient primary brown adipocytes after treatment with cycloheximide was significantly attenuated comparing to that of the WT adipocytes (Fig. 7b), suggesting that the presence of A-FABP accelerates protein degradation of LXRα. Conversely, treatment of cycloheximide together with MG132 (an inhibitor that reduces proteasomal degradation of ubiquitin-conjugated proteins) blocked the degradation of LXRα in both WT and A-FABP-deficient primary adipocytes (Fig. 7c). Furthermore, adenovirus-mediated overexpression of A-FABP significantly enhanced the degradation of LXRα in A-FABP-deficient primary adipocytes comparing with its controls with overexpression of luciferase (Fig. 7d). Taken together, these data suggest that A-FABP inhibits LXRα in part by promoting ubiquitination-dependent proteasomal degradation.

### A-FABP restores T4-induced energy expenditure in KO mice

**Figure 8:**
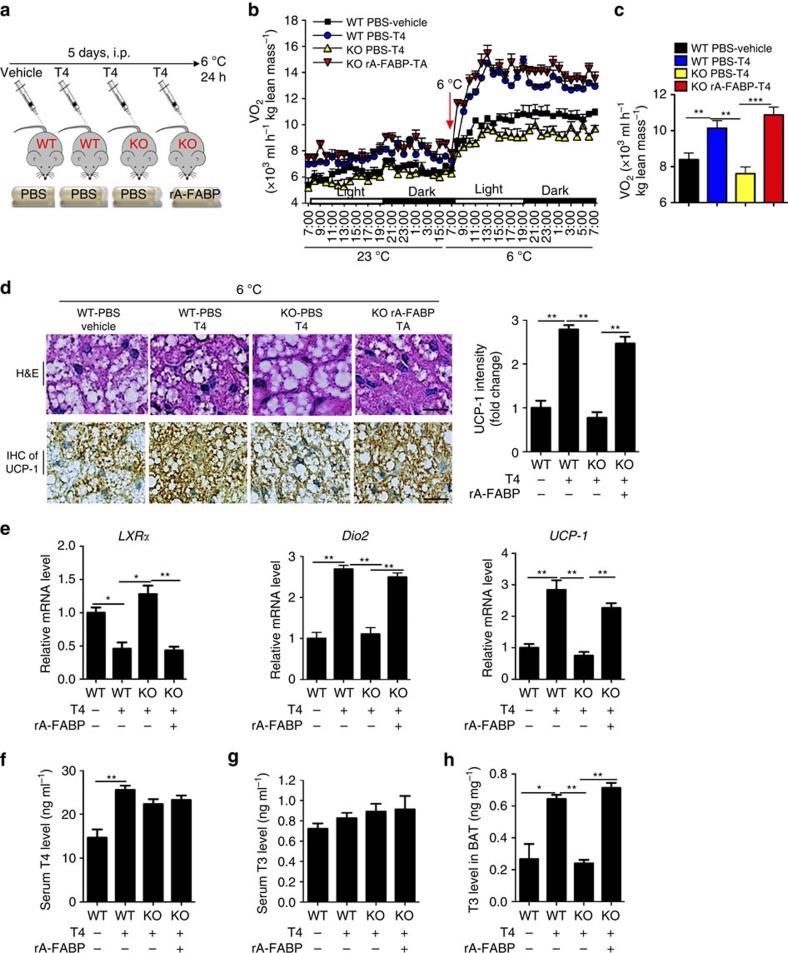
A-FABP enhances conversion of T4 to T3 and energy expenditure in mice. (**a**) Schematic diagram of the experimental procedure. Male 4-week-old A-FABP KO mice and WT littermates fed with HFD for 4 weeks were replenished with rA-FABP (1 μg h^−1^) or PBS for 14 days. Mice were then subcutaneously injected with T4 (400 μg kg^−1^; 5 days) at the last 5 days of recombinant protein administration followed by cold exposure (6 °C) for 24 h (*n=*6). (**b**) Whole-body energy expenditure and (**c**) mean value of cold-induced energy expenditure of mice mentioned above (*n=*6). (**d**) Representative H&E staining, IHC staining and densitometry analysis for the expression of UCP-1 (right panel) in BAT, scale bar, 20 μM, with magnification of 400 × . Representative images from three independent experiments are shown (*n=*6). (**e**) The mRNA abundance of *LXRα, Dio2 and UCP-1* in BAT of above mice (*n=*6). Circulating levels of (**f**) T4 and (**g**) T3 and (**h**) T3 level in BAT of mice mentioned above (*n=*6). Data are represented as mean±s.e.m. *P<0.05, **P<0.01 (one-way analysis of variance with Bonferroni correction for multiple comparisons.)

To confirm the role of A-FABP in intracellular conversion of T4 to T3 in BAT, HFD-fed A-FABP KO and WT mice were infused with either rA-FABP or PBS (as vehicle), followed by subcutaneous injection of T4 for 5 consecutive days and cold exposure for another 24 h (Fig. 8a). Energy expenditure increased significantly in T4-treated WT mice comparing with vehicle-treated controls (Fig. 8b,c). Consistent with the above result (Fig. 5e), the oxygen consumption of T4-treated A-FABP KO mice was comparable to that of WT mice without T4 treatment, while replenishment of rA-FABP together with T4 significantly increased oxygen consumption of A-FABP KO mice similar to that in T4-treated WT mice (Fig. 8b,c), suggesting that A-FABP is essential for the effect of T4 on induction of energy expenditure *in vivo*. Moreover, T4-induced increase in multi-locular cells and expression of UCP-1 in BAT was significantly augmented by infusion of rA-FABP in A-FABP KO mice (Fig. 8d).

In line with our *in vitro* findings (Figs 6 and 7), replenishment with rA-FABP significantly attenuated the expression of *LXRα* in A-FABP KO mice, which was accompanied by enhanced T4-induced expression of *Dio2* and *UCP-1* in BAT (Fig. 8e). Replenishment with rA-FABP did not alter the circulating levels of T4 or T3 in A-FABP KO mice (Fig. 8f,g). However, with the pre-treatment of T4, the intracellular T3 level in BAT of A-FABP KO mice infused with rA-FABP was significantly elevated compared with A-FABP KO mice infused with PBS (Fig. 8h), indicating that A-FABP controls the intracellular conversion of T4 to T3 in BAT. Similar results were also observed in T4-treated WT and A-FABP KO mice with or without replenishment with rA-FABP under room temperature (23 °C). Their body weight, body composition and calorie intake were not altered under these circumstances (Supplementary Fig. 12).

## Discussion

**Figure 9:**
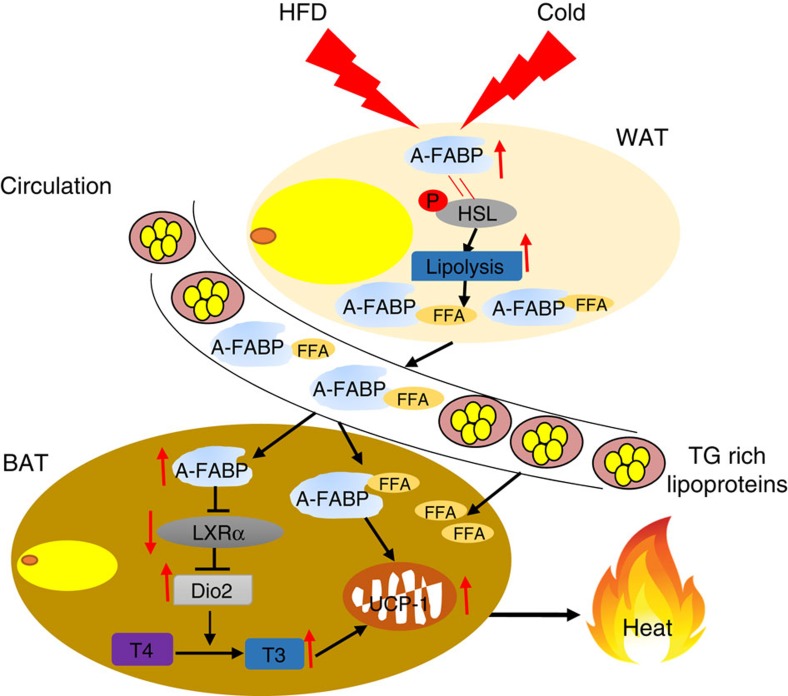
Mechanisms by which A-FABP regulates adaptive thermogenesis. In response to cold challenge or HFD, A-FABP is elevated in BAT, WAT and in the circulation. Elevated A-FABP in BAT induces the expression of *Dio2* via suppression of LXRα. Increased *Dio2* promotes adaptive thermogenesis by enhancing conversion of T4 to T3 in BAT. In addition, elevated circulating A-FABP facilitates the delivery of WAT-derived FFAs to BAT. Increased FFAs supply to BAT further induces the expression and activation of UCP-1 for thermogenesis.

Previous studies showed that A-FABP deficiency exacerbates diet-induced body weight gain in mice. Deletion of A-FABP and E-FABP is also shown to impair thermogenesis in mice in response to cold stress under fasting state^28^. Although the authors speculated that A/E-FABP may have a key role in facilitating FFA transport from circulation to BAT thus providing energy substrate for thermogenesis^28^, this hypothesis was not experimentally validated. Thus, the role of A-FABP and its underlying mechanism in adaptive thermogenesis remains poorly understood. In this study, we demonstrated that A-FABP is a physiological regulator of adaptive thermogenesis in response to both HFD and cold exposure, through its intracellular actions to promote the activation of thyroid hormones and its endocrine actions to transport FFAs released from WAT to BAT for β-oxidation (Fig. 9).

We observed a rapid elevation of A-FABP in bloodstream and adipose tissues in response to cold and HFD challenges. A rapid release of A-FABP from adipose tissues to circulation has also been observed in mice treated with the β_3_-adrenergic receptor agonist CL-316243 (ref. 22), suggesting that A-FABP is an immediate responder of various thermogenic stimuli. While BAT is a main site for adaptive thermogenesis by combustion of FFAs, WAT is a major supply for FFAs through HSL-mediated lipolysis of triglycerides stored in this tissue. Previous studies have demonstrated the role of A-FABP in promoting lipolysis in WAT, by enhancing HSL activity^30^,^36^. However, its function in transport of FFAs is largely ignored, despite the fact that A-FABP is a lipid-binding chaperone. While it is well known that triglyceride-rich lipoproteins are the primary transporters for delivery of FFAs in BAT^37^, we here provided several lines of evidences demonstrating that the stimulatory effects of A-FABP on adaptive thermogenesis are attributed in part to its ability in transporting FFAs released from WAT to BAT. First, the dynamic changes of circulating A-FABP and FFAs levels are strikingly similar in response to thermogenic stimuli (Fig. 3). Second, A-FABP can complex with FFAs in the circulation (Fig. 3). Third, rA-FABP, but not its mutant R126Q which loses the lipid-binding capacity, can directly stimulate the uptake of palmitate in primary brown adipocytes and promote energy expenditure (Fig. 3). Fourth, the defective thermogenesis, impaired BAT activity and reduced FFA uptake into BAT in A-FABP KO mice can be largely reversed by chronic administration of rA-FABP into the circulation (Fig. 4). In line with this notion, capillary endothelial A-FABP is essential for FFA transport from the circulation into FFA-consuming tissues such as heart and skeletal muscle^38^. It is worthy to note that although the abundance of A-FABP is much lower than circulating FFAs, the equilibrium between FFAs and FABP is achieved rapidly within 2 s at 37 °C and within 20 s at 10 °C, and A-FABP possesses the fastest off-rate from its bound FFA comparing with intestinal-FABP and heart-FABP^39^. Therefore, A-FABP may have a complementary role with triglyceride-rich lipoproteins in delivery of FFAs into BAT, by facilitating the release of WAT FFAs into the circulation and transport of FFAs to BAT. Nevertheless, as A-FABP is a cytoplasmic FFA chaperone^20^, it may also facilitate the transportation of FFAs to mitochondria for β-oxidation. In line with our findings, other transporters of FFAs such as CD36 (ref. 40) and fatty acid transport protein 1 (ref. 41), are also important in adaptive thermoregulation by facilitating FFA uptake into BAT.

Furthermore, we uncovered the stimulatory effects of A-FABP on intracellular conversion of T4 to T3 as another important mechanism for promoting adaptive thermogenesis. BAT with abundant expression of both α1 and β1 thyroid hormone receptors is a well-established target of thyroid hormone^42^. The preponderance of thyroid hormones released from the thyroid glands into bloodstream is its inactive form T4, which needs to be converted intracellularly by D2 into its bioactive metabolite T3 for further activation of thyroid hormone receptors^43^. In BAT, thyroid hormones act coordinately with SNS to promote thermogenesis. Upon thermogenic stimuli, activation of SNS induces a marked elevation of D2 expression (approximately 10- to 50-fold) in BAT^7^, which in turn converts T4 to T3 (ref. 44). The elevated T3 further induces D2 expression in BAT^43^, thereby forming a positive feedback loop to increase availability of T3. On the other hand, T3 enhances the expression of β-adrenoceptors in both BAT and WAT^45^, thus potentiating the stimulatory effects of catecholamines on thermogenesis in BAT^6^. Activated thyroid hormone receptors also act directly to increase cAMP-mediated induction of the expression of *UCP-1* gene and to induce the expression of a cluster of genes involved in mitochondrial biogenesis^37^,^46^. Our present study found that thermogenic stimuli (such as cold and HFD challenge)-induced expression of D2 and conversion of T4 to T3 in BAT were markedly decreased in A-FABP KO mice. Furthermore, supplementation of T4 significantly stimulated cold-induced oxygen consumption and BAT activation in WT mice, but not in A-FABP KO mice, although A-FABP KO and WT mice were equally sensitive to norepinephrine-induced BAT activation and thermogenesis. In support of our conclusion, phenotypic changes of D2 KO mice are also strikingly similar to A-FABP KO mice in our study, including diminished conversion of T4 to T3 in BAT, and impaired adaptive thermogenesis in response to cold challenge despite normal serum T3 levels^7^.

Our study demonstrated that the effects of A-FABP on promoting D2 expression and T4 to T3 conversion were mediated by its suppression of LXRα, which is a nuclear receptor having a key role in regulating bile acid, glucose and lipid homeostasis^47^. In BAT of A-FABP KO mice, the impairment in cold-induced expression of D2 was accompanied by an attenuated reduction of LXRα. Vice versa, the effect of LXRα agonist TO901317 on suppression of *Dio2* in primary brown adipocytes was abrogated by treatment with rA-FABP. Activation of LXRα suppresses the transcription of the *Dio2* gene by binding to its promoter, thus reducing D2-mediated T3 production in BAT^32^. In line with our study, LXR α/β −/− mice display increased energy expenditure and UCP-1 expression in BAT while treatment with the LXR agonist GW3965 exerted opposite effects^48^. The expression of *Dio2* is also sixfold higher in the LXRα −/− mice compared with their WT littermates^48^. Furthermore, LXRs form complex with its co-factor, the receptor interacting protein 140, which competes with peroxisome proliferator-activated receptor γ (PPARγ) and peroxisome proliferator-activated receptor gamma coactivator 1-alpha (PGC-1α) for the enhancer region of the *UCP-1* gene promoter and represses the gene expression^49^. Therefore, A-FABP may also enhance UCP-1 expression through its suppressive effect on LXRα.

Consistent with our findings, A-FABP has been shown to downregulate LXRα activity in macrophages, leading to altered *de novo* lipogenesis and ER stress and exacerbated atherosclerosis^33^. Furthermore, A-FABP mediates ubiquitination and the subsequent proteasomal degradation of PPARγ^50^ and LXRα can be ubiquitinated by BRCA1-associated Ring domain/ breast and ovarian cancer susceptibility 1 and its stability and activity can be regulated by ubiquitination-mediated proteasomal degradation^51^ suggesting that A-FABP promotes ubiquitination-dependent proteasomal degradation of LXRα as one of the mechanisms by which A-FABP suppresses LXRα. Further studies are needed to investigate how A-FABP accelerates the proteosomal degradation of LXRα by modulating ubiquitination. Notably, A-FABP is also a downstream target of LXRα activation^52^, suggesting the existence of a negative feedback loop between A-FABP and LXRα. It is also worthy to note that unsaturated FFAs can act as the suppressor of LXRα by preventing its binding to the target genes^53^. As we showed that A-FABP can form complex with circulating FFAs and facilitates their uptake into BAT, it is also possible that A-FABP-mediated accumulation of intracellular unsaturated FFAs interferes with the binding of LXRα to its responsive element on *Dio2* promoter, thereby increasing *Dio2* expression in BAT. In addition, A-FABP can modulate the activity of several transcription factors, including janus kinase 2 and PPARγ^54^,^55^, the latter of which is also a regulator of *LXRα* expression^56^. Further investigations are warranted to explore the involvement of these transcription factors in mediating A-FABP-mediated suppression of LXRα and subsequent induction of the *Dio2* gene in BAT.

While our present study showed the salutary effects of A-FABP on prevention of obesity through promotion of adaptive thermogenesis in BAT, A-FABP actions in other tissues have been shown to exacerbate obesity-related cardiometabolic disorder via its pro-inflammatory activities^18^. In macrophages, A-FABP potentiates toxic lipids- and endotoxin-induced activation of inflammatory pathways (NF-κB and c-Jun N-terminal kinase) and production of pro-inflammatory cytokines^57^. Ablation of A-FABP in macrophage alone is sufficient to render apolipoprotein E deficient mice refractory to spontaneous development of atherosclerosis^58^. The secreted form of A-FABP can also act on endothelium cells to induce endothelial dysfunction^59^, on cardiomyocytes to suppress cardiac contraction^60^ and to mediate cardiac dysfunction during ischemic injury^29^ and on hepatocytes to promote gluconeogenesis leading to altered glucose homeostasis^22^. On the other hand, circulating A-FABP may possess insulinotropic action mediating glucose-stimulated insulin secretion of pancreatic β cells^61^. Therefore, these findings, together with our present study, highlight the complex functions of A-FABP in obesity and its associated cardiometabolic disorders due to its differential effects on various target tissues at different stages of the disease. It is likely that elevated A-FABP under physiological stimuli (such as cold challenge) or early phase of obesity may serve as a defense response to promote adaptive thermogenesis through its actions in adipocytes. However, with the progression of obesity, prolonged and excessive increase of A-FABP may exacerbate metabolic and cardiovascular disorders through its effects on non-adipose tissues, including macrophages, endothelium, cardiomyocytes and hepatocytes. The dual effects of A-FABP on obesity and its associated medical complications are strikingly reminiscent of leptin, another adipocyte-secreted hormone that is elevated in both animals and human with obesity^62^,^63^. Intriguingly, leptin combats obesity through its hypothalamic actions to reduce food intake and its actions in brown adipocytes to enhance energy expenditure via promoting D2 activity and conversion of T4 to T3 in BAT^64^. In contrast, excessive elevation of leptin can also cause cardiovascular diseases, liver inflammation and fibrosis through its actions on blood vessels, cardiomyocytes and liver^65^,^66^.

In conclusion, our study demonstrated an important role of adipocyte-derived A-FABP in adaptive thermogenesis, via its actions on conversion of T4 to T3 by modulating the LXRα-*Dio2* signalling axis and facilitating the uptake of circulating FFAs into BAT. Notably, the pharmacological inhibitors of A-FABP such as BMS309403 (ref. 67) and the recent identified A-FABP monoclonal antibody CA33 (ref. 68) alleviate metabolic and cardiovascular disorders in animals. Our study suggests that global pharmacological inhibition of A-FABP may not be an optimal therapeutic strategy for obesity-related cardiovascular and metabolic diseases due to the potential impairment of adaptive thermogenesis. Further investigations to dissect the structural and molecular basis underlying the differential effects of A-FABP in different tissues are needed in order to design more effective therapeutic interventions for obesity and its related medical complication by targeting A-FABP.

## Methods

### Animals

A-FABP KO mice in C57BL/6N background were generated using the same procedures as previously described^29^. Age-matched male A-FABP KO mice and their littermates were used in all the experiments of this study. Animals were allocated to their experimental group according to their genotypes. No randomization of mice was used. The investigators were not blinded to the experimental groups. Mice were housed in a temperature-controlled facility (23 °C, 12-hour light/dark cycle, 60–70% humidity). Four-week-old mice were weaned and fed with either STC (Purina, Framingham, MA, USA) or Western diet (D12079B, Research Diet, USA) for 4 or 24 weeks. Body composition was determined bi-weekly by nuclear magnetic resonance (Bruker, minispec, Germany). All experimental protocols were approved by the Committee on the Use of Live Animals in Teaching and Research at the University of Hong Kong.

### Cold exposure

Male 4-week-old A-FABP KO mice and their littermates fed with STC or HFD for 24 weeks were provided with food and water *ad libitum* at 6 °C for 8 or 24 h. Rectal temperature was measured with 4610 Precision Thermometer (Thermo Scientific, MA, USA), and serum was collected at various time points via tail vein for lipid profile analysis.

### Glucose tolerance test and insulin tolerance test

For glucose tolerance test, male 4-week-old A-FABP KO mice and their littermates fed with STC or HFD for 24 weeks mice were housed in clean cages with fasting for 16 h before intra-peritoneally injected with D-glucose (1 g kg^−1^). Blood glucose was monitored at 0, 10, 20, 30, 45, 60, 75 and 90 min after glucose injection. Male 4-week-old A-FABP KO mice and their littermates fed with HFD for 4 weeks infused with rA-FABP (1 μg h^−1^) or its mutant R126Q (1 μg h^−1^) were also subjected to glucose tolerance test. For insulin tolerance test, male 4-week-old A-FABP KO mice and their littermates fed with STC or HFD for 24 weeks mice were fasted for 6 h followed by intra-peritoneal injection of insulin (1 U kg^−1^ for mice fed with HFD and 0.5 U kg^−1^ for mice fed with STC). Blood glucose was measured at 0, 20, 40, 60 and 80 min after insulin injection. HOMA index was calculated according to the formula: fasting insulin (micro U l^−1^) × fasting glucose (nmol l^−1^)/22.5.

### Indirect calorimetry

Whole-body oxygen consumption (VO_2_) was assessed using Indirect Calorimetry with the Columbus Comprehensive Lab Animal Monitoring System (CLAMS, Columbus, USA) as previously described^12^. Briefly, male 4-week-old A-FABP KO mice and their littermates fed with STC or HFD for 4 weeks, mice were acclimated to CLAMS cages individually with food and water *ad libitum* for 24 h. Data on OCR (VO_2_) were recorded every 10 or 11 min for a 48-hour period at 23 °C or 6 °C. Physical activity was measured by infrared technology (OPT-M3, Columbus Instruments). Norepinephrine-induced energy expenditure was determined. Briefly, male 4-week-old A-FABP KO mice and their littermates fed with STC or HFD for 24 weeks mice were anaesthetized and VO_2_ was recorded for the first 30 min to access basal energy expenditure. Individual mice were then injected with norepinephrine (1 mg kg^−1^, Sigma-Aldrich), and VO_2_ was determined for another 60 min.

### Generation of rA-FABP and A-FABP mutant R126Q

Mouse A-FABP (GenBank BC054426.1) was cloned into the His-tag expression vector pDEST17. A-FABP mutant R126Q was generated using Quick Change Multi Site-Directed Mutagenesis kit (Life Technology, USA) with pDEST17-A-FABP vector, using the primers described in Supplementary Table 1. Vectors were transformed into BL21 *Escherichia coli*, induced with isopropyl β-D-1-thiogalactopyranoside (1 mmol l^−1^; Sigma-Aldrich), and His-tagged A-FABP was purified with imidazole (Sigma-Aldrich). Identity and purity of protein were confirmed by SDS–polyacrylamide gel electrophoresis, followed by Western blot or Coomassie blue staining. Endotoxin was removed by Pierce High Capacity Endotoxin Removal Spin Columns (Thermo Fisher Scientific, USA) and measured by QCL-1000 End point Chromogenic LAL Assays (Lonza, Switzerland, value<0.02 EU μg^−1^ protein).

### Generation of adenovirus expressing A-FABP and luciferase

Adenovirus over-expressing A-FABP (Ad-AFABP) was generated using AdEasy XL Adenoviral Vector System according to the manufacturer's instruction (BD Biosciences, USA). Briefly, mouse A-FABP (GenBank BC054426.1) was cloned to pShuttle2 vector followed by ligating the expression cassette to BD Adeno-XTM Viral DNA. Recombinant Ad-AFABP was propagated in HEK293 cells and purified using AdEasy Virus Purification Kits (Stratagene, La Jolla, California, USA). Recombinant adenovirus encoding luciferase (Ad-Luci) was kindly provided by Christopher Rhodes (University of Washington, Seattle)^69^. Briefly, the recombinant virus was packaged and amplified in HEK293 cells and purified by cesium chloride density gradient centrifugation.

### Isolation and culture of primary adipocytes

The Stromal vascular fractions (SVFs) of BAT were isolated as previously described^12^ with modification. Freshly isolated BAT pads from male 6-week-old A-FABP KO mice and their relative WT littermates were minced and digested in 0.1% (w/v) collagenase type I (Invitrogen, CA, USA) for 30 min at 37 °C with gentle shaking in water bath. The digestion mixture was passed through a 70 μm cell strainer (BD Biosciences) and centrifuged at 700*g* for 5 min at 4 °C. The SVFs were cultured in Dulbecco's Modified Eagle Medium (DMEM) with 10% fetal bovine serum (Invitrogen, CA, USA) and 1% penicillin and streptomycin (PSF, Thermo Fisher Scientific, USA) until confluence. To differentiate SVFs into mature adipocytes, SVFs cells were treated with insulin (20 nM, Novartis, Swiss), 3-isobutyl-1-methylxanthine (0.5 mM, Sigma-Aldrich), dexamethasone (1 μM, Sigma-Aldrich), 3,3,5-triiodothyronine (T3, 1 nM, Sigma-Aldrich), indomethacin (125 nM, Sigma-Aldrich), retinoic acid (20 μM, Sigma-Aldrich) and Vitamin C (142 μM, Sigma-Aldrich) in DMEM for 2 days then changed to DMEM containing insulin (20 nM) and T3 (1 nM) for another 4 days.

### Measurement of cellular OCR

Cellular OCR was measured using the XFe24 Extracellular Flux Analyser (Seahorse Bioscience, USA). SVFs isolated from male 6-week-old A-FABP KO mice and their relative WT littermates were seeded in XFe24-well microplate with 1.5 × 10^4^ cells per well and differentiated into mature adipocytes. Cells were pre-incubated with BSA (3 μg ml^−1^), rA-FABP (2 μg ml^−1^) or palmitate (200 nM, Sigma-Aldrich) for 30 min. Oligomycin (5 μM, ATP synthase inhibitor, Sigma-Aldrich), carbonyl cyanide-4-(trifluoromethoxy) phenylhydrazone (FCCP, 50 μM, cellular uncoupler, Sigma-Aldrich), rotenone/antimycin A (1 μM, Sigma-Aldrich) were sequentially added to determine basal-, ATP-dependent-, maximal- and mitochondria-independent oxygen consumption, respectively.

### Replenishment of rA-FABP and thyroid hormones

Thyroxine (T4, 400 μg kg^−1^, 5 days, Sigma-Aldrich) or T3 (500 μg kg^−1^, 1 day, Sigma-Aldrich) were administrated into male 4-week-old A-FABP KO mice and their WT littermates fed with HFD for 4 weeks by subcutaneous injection. The rA-FABP or A-FABP mutant R126Q was infused into the male 4-week-old A-FABP KO mice and their WT littermates fed with HFD for 4 weeks by subcutaneous implantation of ALZET Osmotic Pumps (Model 2004, Alzert, Cupertino, CA, USA) at a constant rate of 1 μg h^−1^ for 14 days. The circulating levels of A-FABP, T3 and T4 were measured with their respective immunoassays.

### Palmitate uptake in mice and primary adipocytes

Male 8-week-old A-FABP KO mice and their relative WT littermates infused with rA-FABP (1 μg h^−1^) or R126Q (1 μg h^−1^) or PBS for 14 days were orally administrated with 200 μl olive oil containing ^3^H-palmitate (2 μCi, PerkinElmer, USA) for 4 h in the absence of food and water. Interscapular BAT, subcutaneous WAT and epididymal WAT and various peripheral tissues were freshly isolated and minced for measurement of radioactivity. For *in vivo* FA tracing, BODIPY-FA (20 μM) was pre-incubated with or without rA-FABP (50 μg) or A-FABP mutant R126Q (50 μg) for 30 min. These BODIPY-FAs were injected through tail vein into male 8-week-old A-FABP KO mice, and the fluorescence was monitored by the PE IVIS Spectrum *in vivo* imaging system (PerkinElmer, USA). For palmitate uptake in primary adipocytes, ^3^H-palmitate (55 mCi mmol^−1^, PerkinElmer, USA) or BODIPY-FA (2 μM, Molecular Probes) was pre-incubated with BSA (3 μg ml^−1^) or rA-FABP (2 μg ml^−1^) 30 min before adding to differentiated primary adipocytes for 10 min. Radioactivity or fluorescence of cell lysates was measured by Liquid Scintillation Counter (PerkinElmer, USA) or Infinite M200 Microplate Reader (Tecan Systems, Inc. San Jose, CA, USA), respectively. For tracing of BIODIPY-FA and A-FABP, rA-FABP was fluorescent-labelled using Alexa Fluor 488 Protein Labelling Kit (Invitrogen, CA, USA). BODIPY-FA (2 μM) was pre-incubated with or without fluorescent-labelled rA-FABP (2 μg ml^−1^) for 30 min, and was then added to A-FABP-deficient primary brown adipocytes. The fluorescent images of cells were obtained at different time points using a microscope (Bx41 System, Olympus) with a colour digital camera (Olympus Model DP72).

### Co-immunoprecipitation of A-FABP and ^3^H-palmitate

One-hundred microlitre of serum of male 8-week-old C57BL/6N mice orally administrated with ^3^H-palmitate (2 μCi, PerkinElmer, USA) was harvested after 4 h, followed by immunoprecipitation with goat anti-mouse/rat A-FABP antigen affinity-purified polyclonal antibody (5μg ml^−1^, AF1443, R&D Systems, Minneapolis, USA,) or anti-mouse IgG (5 μg ml^−1^, 02–6502, Thermo Fisher Scientific, USA) at 6 °C overnight. The immunocomplexes were precipitated by incubation with protein G beads (10,003, Thermo Fisher Scientific, USA) at 23 °C for 2 h. After washing with ice-cold lysis buffer for six times, the immunoprecipitated complexes were subjected to either Western blot analysis after eluted protein from 20 μl of beads by heating in 20 μl of 2 × SDS loading buffer for 10 min at 50 °C or liquid scintillation counting to determine the radioactivity of ^3^H-palmitate^70^.

### ^14^C-glucose uptake

Male 4-week-old A-FABP KO mice and their WT littermates fed with HFD for 4 weeks were intra-peritoneally injected with 2-[1-^14^C]-deoxy-D-glucose (20 μCi, PerkinElmer, USA) for 2 h in the absence of food and water. Interscapular BAT, liver and soleus muscle were isolated and minced for measurement of radioactivity and normalized with protein concentrations.

### Immunoblot analysis and real-time PCR

Proteins were separated by SDS–polyacrylamide gel electrophoresis, transferred to polyvinylidene difluoride membranes, and probed with primary antibodies A-FABP (0.25 μg ml^−1^, goat polyclonal; AF1443, R&D Systems), UCP-1 (0.5 μg ml^−1^, rabbit polyclonal; ab10983, Abcam, Cambridge, UK), liver X receptor α (LXRα, 1 μg ml^−1^, rabbit monoclonal; ab28478, Abcam), type II iodothyronine deiodinase (D2, 0.25 μg ml^−1^, rabbit polyclonal; ab77481, Abcam), tyrosine hydroxylase (0.25 μg ml^−1^, rabbit polyclonal; 2,792, Cell Signaling), β-tubulin (0.25 μg ml^−1^, rabbit polyclonal; 2,128, Cell Signaling, Beverly, MA, USA) and glyceraldehyde 3-phosphate dehydrogenase (GAPDH, 0.1 μg ml^−1^, rabbit monoclonal; 5,174, Cell Signaling). The intensities of protein bands were quantified using the NIH Image J software.

Total RNA was extracted with Trizol (Invitrogen) and reverse transcribed into complementary DNA using Improm-II reverse transcription kit (Promega, Madison, USA). Real-time PCR was performed using SYBR Green master mix (Qiagen, Venlo, the Netherlands) on a 7,900 HT (Applied Biosystems, CA, USA), normalized against the GAPDH gene. Primer sequences are listed in Supplementary Table 1.

### Analysis of mRNA stability in primary brown adipocytes

SVFs isolated from male 6-week-old A-FABP KO mice and their relative WT littermates were differentiated into primary brown adipocytes in 12-well plates. Differentiated adipocytes were treated with actinomycin D (actD, 1 μg ml^−1^) or vehicle (PBS). The mRNA abundance of *LXRα* was measured at various time points (0, 4, 6, 12 h) by real-time PCR.

### Analysis of protein stability in primary brown adipocytes

SVFs isolated from male 6-week-old A-FABP KO mice and their WT littermates were differentiated into primary brown adipocytes in 12-well plates. WT and A-FABP-deficient primary adipocytes were treated with cycloheximide (50 μg ml^−1^, Sigma-Aldrich) in the presence or absence of the proteasome inhibitor MG132 (10 μM, Sigma-Aldrich). In addition, A-FABP deficient primary adipocytes were infected with adenovirus over-expressing A-FABP (Ad-AFABP) or luciferase (Ad-Luci) at fifty multiplicity of infection (M.O.I) for 48 h followed by treatment with cycloheximide (50 μg ml^−1^). Adipocytes were harvested at indicated time points and the expression of LXRα and A-FABP was determined by western blot analysis.

### Histological and immunohistochemistry analysis

Paraffin-embedded adipose tissues were prepared at the thickness of 5 μM. Deparaffinized and dehydrated sections were stained with haematoxylin and eosin (Sigma-Aldrich) as previously described^29^. For immunocytochemistry, sections were sequentially incubated with primary antibody UCP-1 (5 μg ml^−1^, rabbit polyclonal; Abcam, UK) overnight and anti-rabbit secondary antibody (4 μg ml^−1^; Cell Signaling Technology) for 1 h at 23 °C, followed by development with 3, 3′ diaminobenzidine solution (Sigma-Aldrich). The nuclei were counter-stained with haematoxylin. The intensities of positively stained cells were quantified in each of five randomly selected fields by the Image J software. Two independent investigators blinded to sample identity, one investigator performed the staining and another investigator analysed the adipose tissue sections.

### Biochemical and immunological analysis

Serum insulin, adiponectin and A-FABP levels were measured using Advanced Ultra Sensitive Mouse Insulin Immunoassay kit, mouse adiponectin ELISA kit (AIS, HKU, Hong Kong) and mouse A-FABP ELISA kit (BioVendor Laboratory Medicine, Modrice, Czech Republic) respectively. T4 and T3 levels in serum or adipose tissues were analysed using mouse T4 or T3 ELISA kit, respectively (Calbiotech, Spring Valley, CA, USA). Plasma glucose was measured using an ACCU-Check glucose meter (Roche, Indianapolis, IN, USA). Serum FFAs, triglyceride and cholesterol were determined using FFAs, Half Micro Test kit (Roche, USA), Stanbio Liquicolor Triglyceride and Stanbio Liquicolor Cholesterol (STANBIO Laboratory, USA), respectively.

### Statistical analysis

All statistical analyses were performed using Prism 6 (GraphPad Software Inc. La Jolla, CA92037 USA). Data were expressed as mean±s.e.m. Animal sample size for each study was chosen on the basis of literature documentation of similar well-characterized experiments, and no statistical method was used to predetermine sample size. Statistical significance was assessed by Student's *t*-test or one-way analysis of variance with Bonferroni correction for multiple comparisons. A value of *P<*0.05 was considered statistically significant. Statistical outlier analysis was calculated using the GraphPad Outlier calculator (http://graphpad.com/quickcalcs/Grubbs1.cfm). Those significant outliers were excluded from data analysis.

### Data availability

The data supporting the findings of this study are available within the article and its Supplementary Information Files, or are available from the corresponding author upon reasonable request.

## Additional information

**How to cite this article:** Shu, L. *et al*. A-FABP mediates adaptive thermogenesis by promoting intracellular activation of thyroid hormones in brown adipocytes. *Nat. Commun.* **8,** 14147 doi: 10.1038/ncomms14147 (2017).

**Publisher's note:** Springer Nature remains neutral with regard to jurisdictional claims in published maps and institutional affiliations.

## Supplementary Material

Supplementary Information Supplementary Figures, Supplementary Table.
