# Supplementary material for: A-FABP mediates adaptive thermogenesis by promoting intracellular activation of thyroid hormones in brown adipocytes
Source: Nat Commun. 2017 Jan 27;8:14147. doi: 10.1038/ncomms14147 (PMC5290165; doi:10.1038/ncomms14147)
Supplement: Supplementary Information — Supplementary Figures, Supplementary Table. [file ncomms14147-s1.pdf]

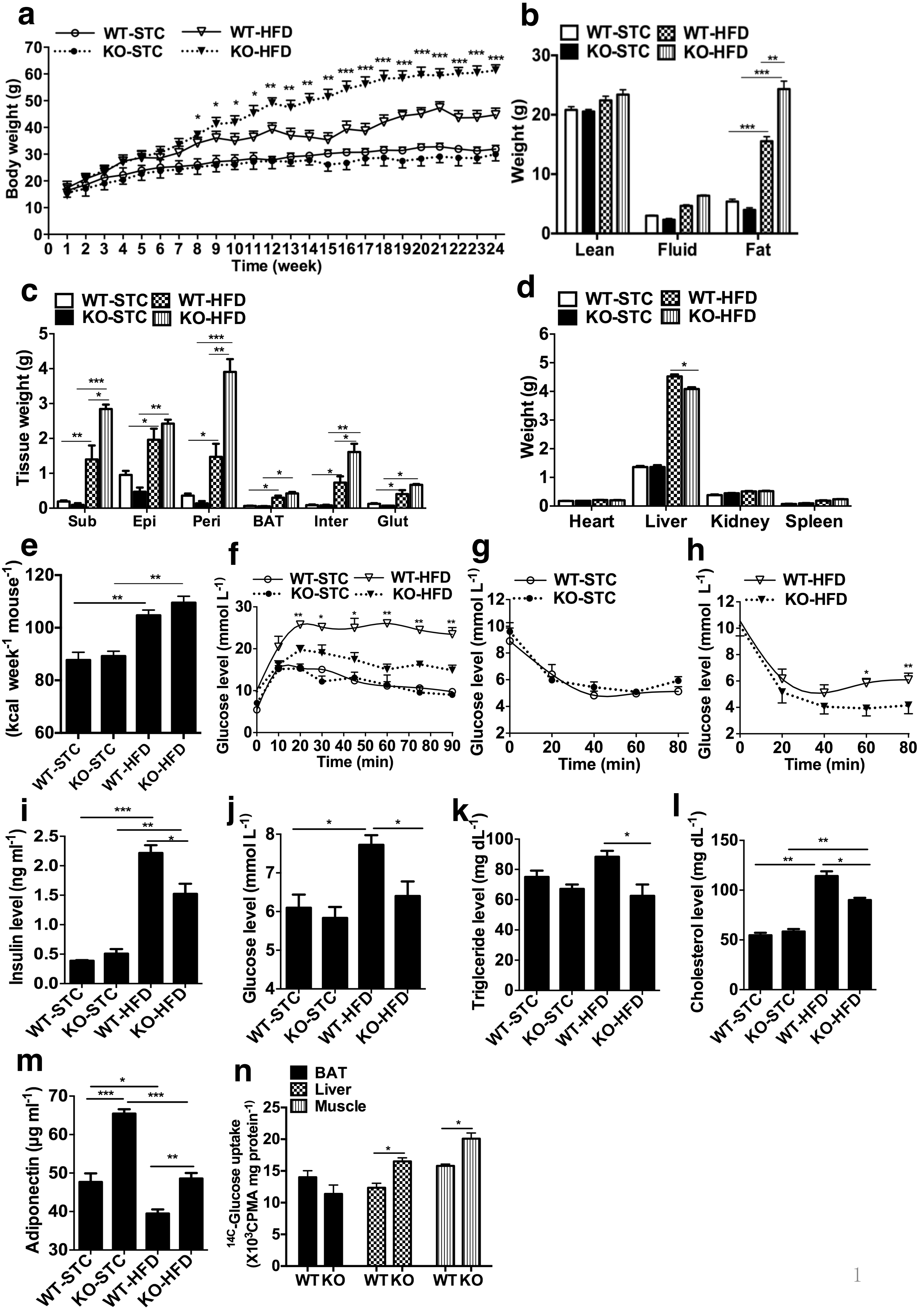
a
WT-STC
KO-STC
WT-HFD
KO-HFD
Body weight (g)
Time (week)
b
Weight (g)
Lean
Fluid
Fat
c
Tissue weight (g)
Sub
Epi
Peri
BAT
Inter
Glut
d
Weight (g)
Heart
Liver
Kidney
Spleen
e
Calorie intake (kcal week-1 mouse-1)
f
Glucose level (mmol L-1)
Time (min)
g
h
i
Insulin level (ng ml-1)
j
Glucose level (mmol L-1)
k
Triglceride level (mg dL-1)
l
Cholesterol level (mg dL-1)
m
Adiponectin (µg ml-1)
n
BAT
Liver
Muscle
14C-Glucose uptake (X10³CPMA mg protein-1)
WT KO

Supplementary Figure 1. Basic parameters of A-FABP KO mice and their WT littermates fed with STC or HFD for 24 weeks. **(a)** Body weight, **(b)** body composition, **(c)** weight of fat tissues (Sub, subcutaneous; Epi, epididymal; Peri, peri-renal; BAT, brown adipose tissue; Inter, Interscapular; Glut, gluteal) and **(d)** various organs as specified, **(e)** average calorie intake, **(f)** glucose tolerance test (GTT) and (**g-h**) insulin tolerance test (ITT) of the mice, **(i)** fasting serum insulin, **(j)** fasting glucose, **(k)** triglyceride, **(l)** cholesterol, and **(m)** adiponectin levels of male 4-week-old A-FABP KO mice and their WT littermates fed with STC or HFD for 24 weeks (*n*=12). **(n)** 2-[1-^{14}C]-Deoxy-D-glucose uptake in BAT, liver and soleus muscle of male 4-week-old A-FABP KO and their WT littermates fed with HFD for 4 weeks *(n=6)*. Data are represented as mean ± s.e.m. **p<0.05, **p<0.01, ***p<0.001* (Students' t-test (h,n), one-way ANOVA with Bonferroni correction for multiple comparisons (a-f, i-m)).

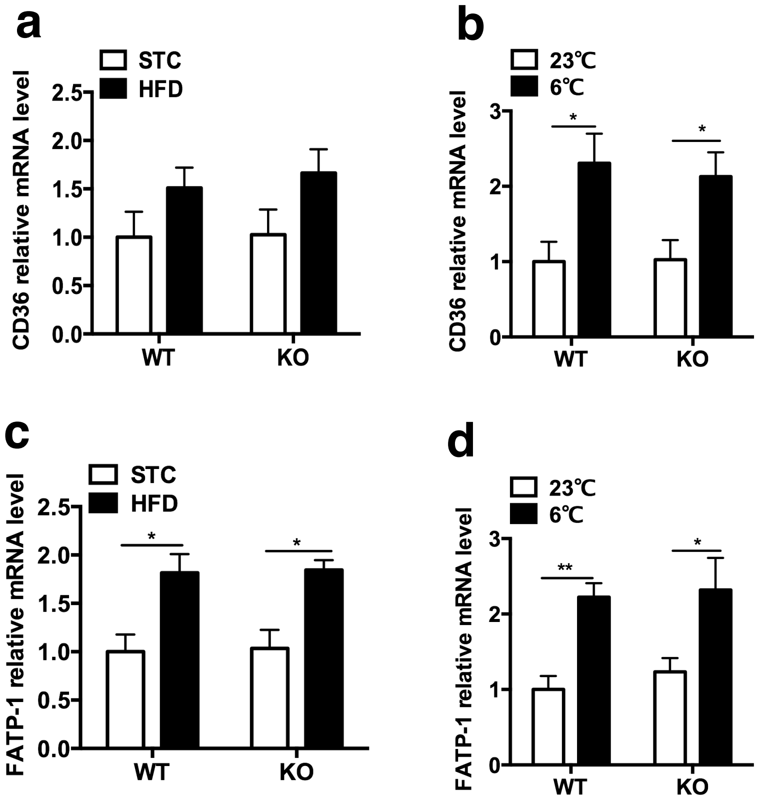

Supplementary Figure 2. A-FABP deficiency does not impair the expression of the membrane fatty acid transporters in BAT of mice. Male 4-week-old A-FABP KO mice and their WT littermates were fed with either **(a, c)** STC or HFD for 24 weeks or **(b, d)** subjected to room temperature (23 °C) or cold exposure (6 °C) for 24 hours *(n=6)*. The mRNA abundance of **(a-b)** *CD36* and **(c-d)** *FATP-1* in BAT of WT and A-FABP KO mice was determined by real-time PCR *(n=6)*. Data are represented as mean ± s.e.m. **p<0.05, **p<0.01* (Students' t-test).

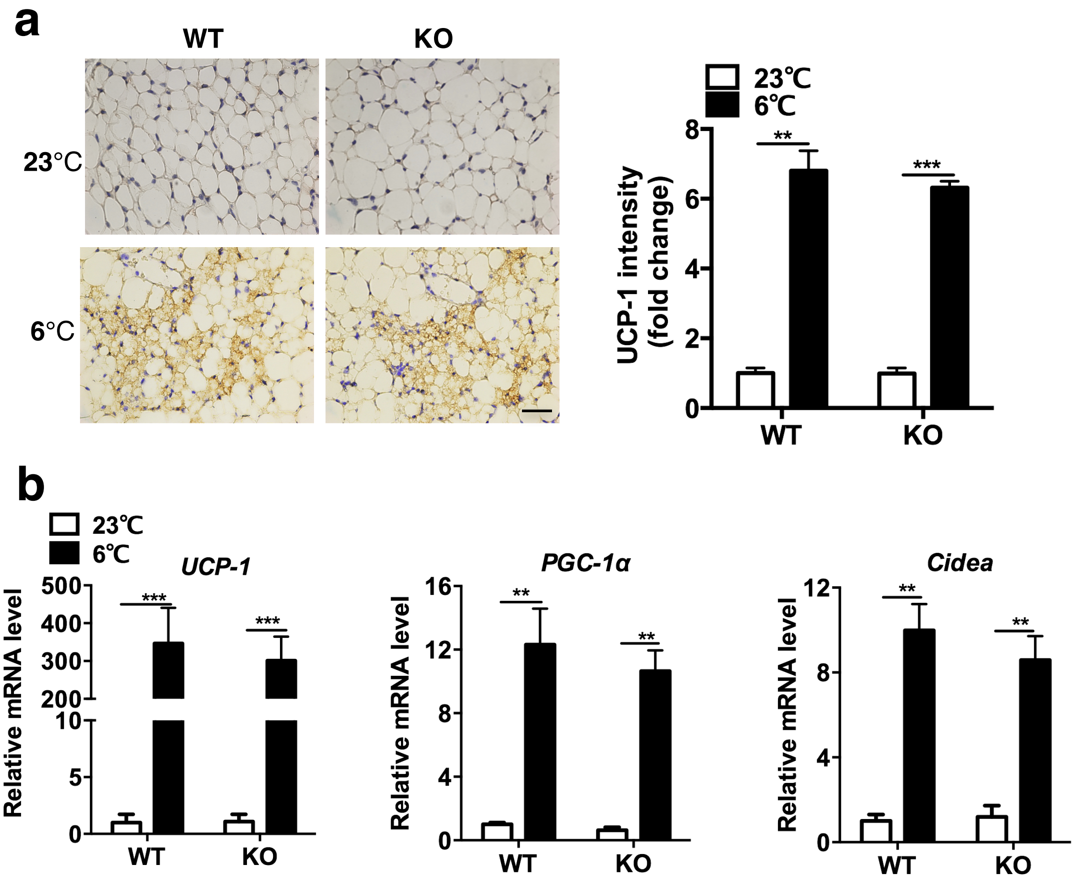

Supplementary Figure 3. A-FABP deficiency does not impair the browning program in subcutaneous fat of mice. Male 8-week-old A-FABP KO mice and their WT littermates on STC were subjected to room temperature (23 °C) or cold exposure (6 °C) for 24 hours. **(a)** Representative IHC staining of UCP-1 in subcutaneous fat, scale bar=20 μm; with magnification of 400x. The right panel is the densitometry analysis for UCP-1. Representative images from three independent experiments are shown *(n=6).* **(b)** The mRNA abundance of *UCP-1*, *PGC-1α* and *Cidea* in subcutaneous fat of above WT and A-FABP KO mice after cold exposure for 24 hours was determined by real-time PCR *(n=6)*. Data are represented as mean ± s.e.m. ***p<0.01, ***p<0.001* (Students' t-test).

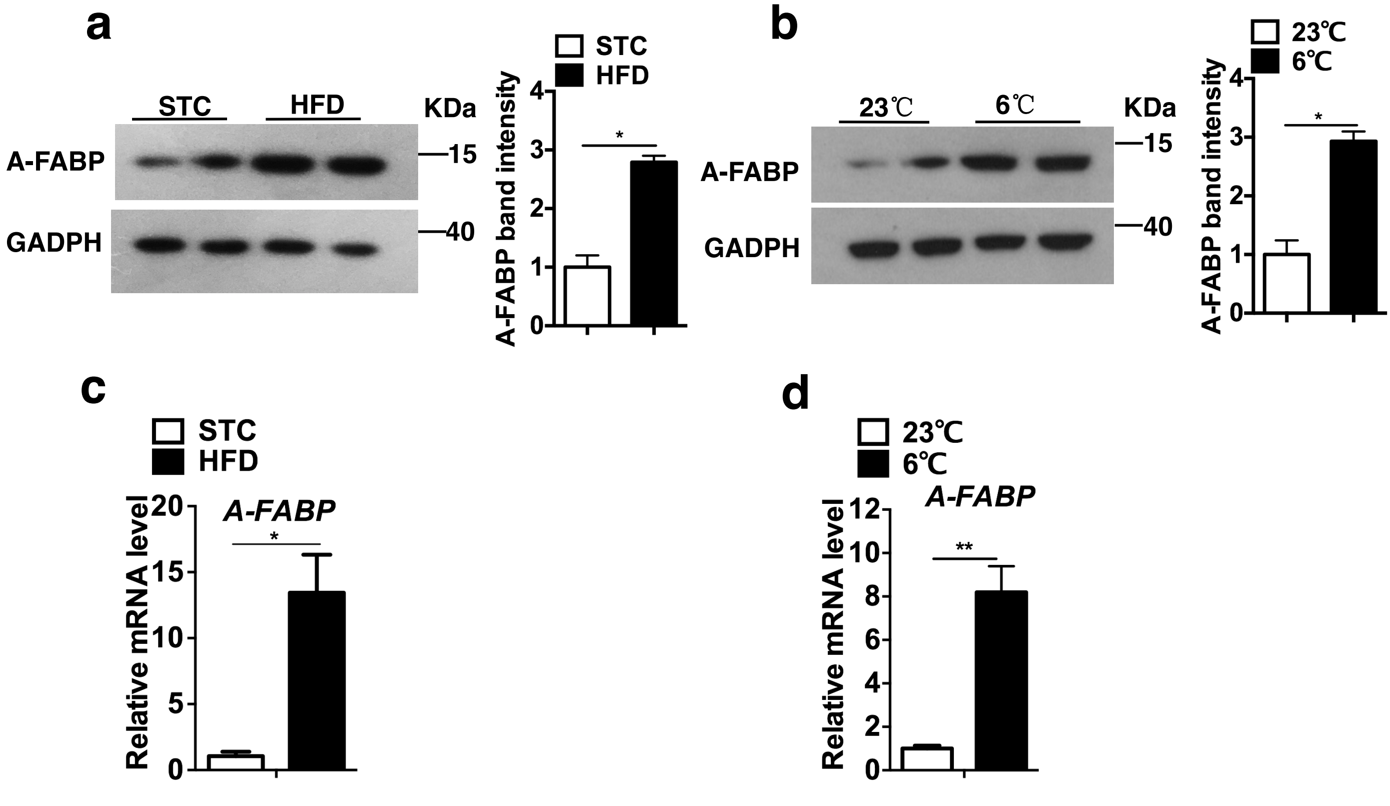

Supplementary Figure 4. Expression of A-FABP is elevated in epididymal fat of C57BL/6N mice in response to thermogenic stimuli. (a-b) Epididymal fat isolated from **(a)** male 4-week-old C57BL/6N mice fed with STC or HFD for 24 weeks or **(b)** male 8-week-old C57BL/6N mice subjected to room temperature (23 °C) or cold exposure (6 °C) for 24 hours were subjected to immunoblotting using an antibody against A-FABP, GADPH as indicated. The bar charts in the right panel are the band intensity of A-FABP relative to GADPH and expressed as arbitrary units *(n=6)*. **(c-d)** The mRNA abundance of *A-FABP* in epididymal fat of above C57BL/6N mice **(c)** fed with STC or HFD for 24 weeks or **(d)** subjected to room temperature (23 °C) or cold exposure (6 °C) for 24 hours was determined by real-time PCR *(n=6)*. Uncropped western blot images are shown in Supplementary Fig. 15. Data are represented as mean ± s.e.m. **p<0.05, **p<0.01* (Students' t-test).

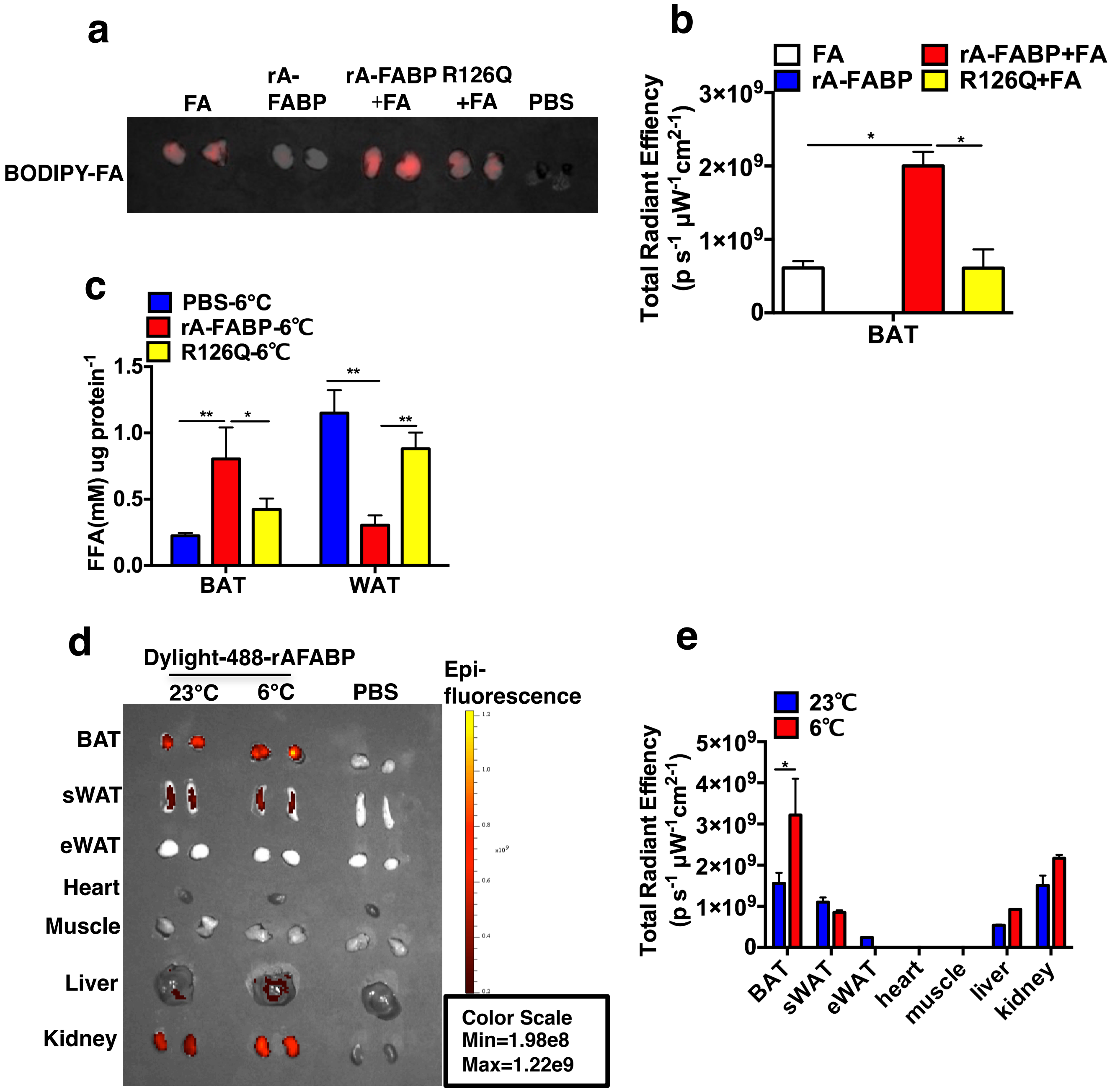

Supplementary Figure 5. Exogenous A-FABP facilitates FFA uptake in brown adipose tissue (BAT) and is mainly destined to BAT in response to cold exposure in mice. (a-b) Male 8-week-old A-FABP KO mice were intravenously injected with BODIPY-FA (20 μM) pre-incubated with or without recombinant A-FABP (rA-FABP; 50 μg) or its mutant R126Q (50 μg). **(a)** *In vivo* fluorescence imaging analysis of BAT at 1 hour after injection. **(b)** Quantification of fluorescence intensity in BAT *(n=4).* **(c)** FFA level in BAT and WAT of A-FABP KO mice infused with PBS, rA-FABP (1 μg $hour^{-1}$) or R126Q (1 μg $hour^{-1}$) for 14 days followed by cold exposure (6°C) for 8 hours *(n=4).* **(d-e)** A-FABP KO mice were intravenously injected with fluorescent-labeled rA-FABP (50 μg) and subjected to room temperature (23°C) or cold exposure (6°C) for 1 hour. **(d***)* *In vivo* fluorescence imaging analysis of various tissues. (**e**) Quantification of fluorescence intensity in various tissues *(n=4).* Data are represented as mean ± s.e.m. *$^*p<0.05$, $^{**}p<0.01$* (One-way ANOVA with Bonferroni correction for multiple comparisons).

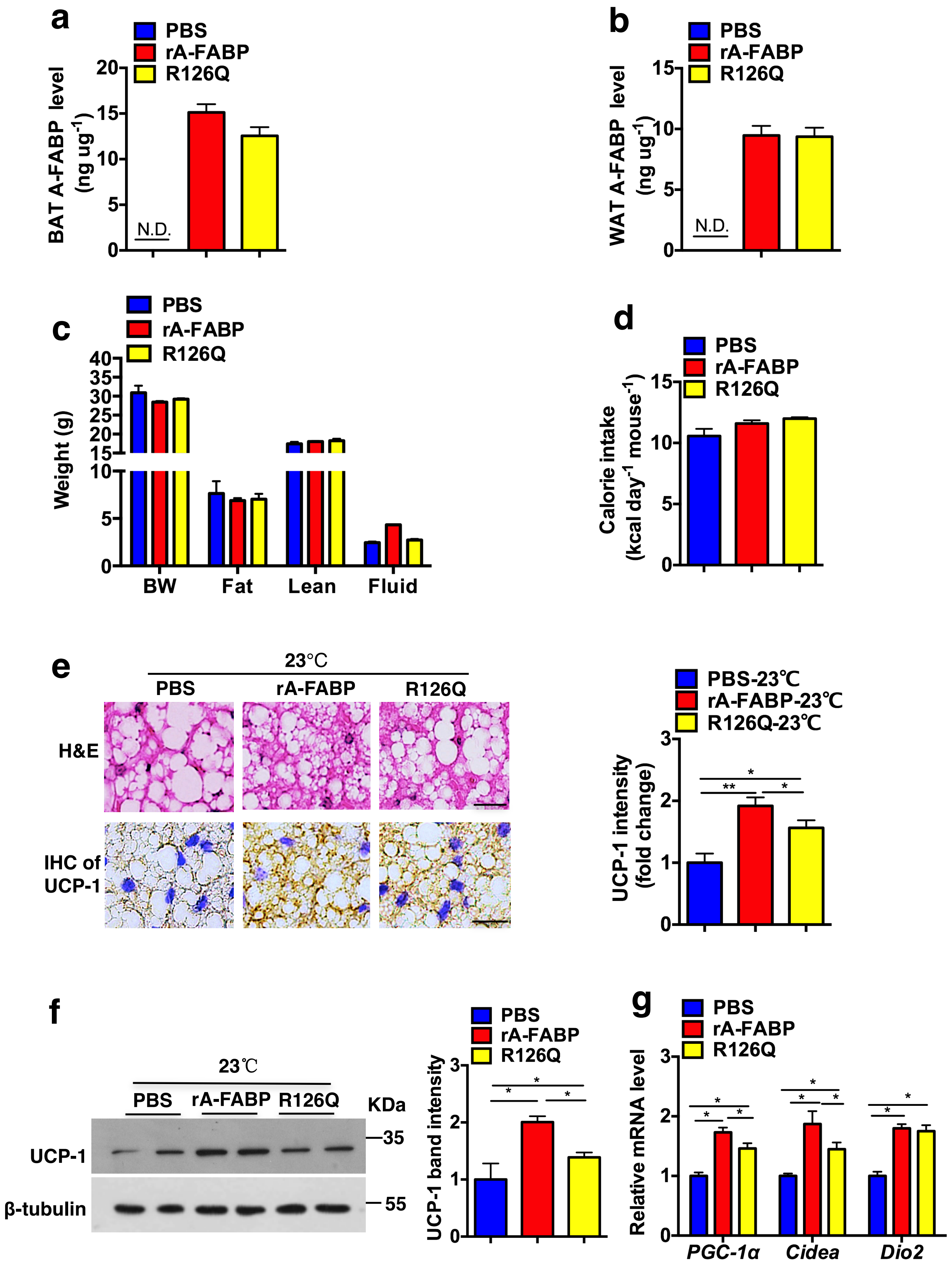
a
PBS
rA-FABP
R126Q
BAT A-FABP level
(ng ug-1)
20
15
10
5
0
N.D.
b
PBS
rA-FABP
R126Q
WAT A-FABP level
(ng ug-1)
15
10
5
0
N.D.
c
PBS
rA-FABP
R126Q
Weight (g)
40
35
30
25
20
15
10
5
0
BW
Fat
Lean
Fluid
d
PBS
rA-FABP
R126Q
Calorie intake
(kcal day-1 mouse-1)
15
10
5
0
e
23°C
PBS
rA-FABP
R126Q
H&E
IHC of
UCP-1
PBS-23℃
rA-FABP-23℃
R126Q-23℃
UCP-1 intensity
(fold change)
3
2
1
0
*
**
*
f
23℃
PBS
rA-FABP
R126Q
KDa
UCP-1
β-tubulin
35
55
PBS
rA-FABP
R126Q
UCP-1 band intensity
3
2
1
0
*
*
*
g
PBS
rA-FABP
R126Q
Relative mRNA level
3
2
1
0
*
*
*
*
*
*
*
*
PGC-1α
Cidea
Dio2

Supplementary Figure 6. Replenishment of recombinant A-FABP leads to a modest elevation in energy expenditure and BAT recruitment in A-FABP KO mice at 23°C. Male 4-week-old A-FABP KO mice fed with HFD for 4 weeks were infused with PBS, recombinant A-FABP (rA-FABP, 1μg hour^{-1}) or A-FABP mutant R126Q (1 μg hour^{-1}) and housed at 23 °C for 14 days. **(a-b)** A-FABP level in **(a)** BAT and **(b)** WAT of A-FABP KO mice after infusion of recombinant proteins *(n=4).* **(c)** Body weight, body composition and **(d)** calorie intake of the mice *(n=4).* **(e)** Representative H&E staining and IHC staining of UCP-1 in BAT of the mice, scale bar =20 μm; with magnification of 400x. The right panel is the densitometry analysis for UCP-1. Representative images from three independent experiments are shown *(n=4).* **(f)** BAT isolated from above A-FABP KO mice were subjected to immunoblotting using an antibody against UCP-1 and β-tubulin as indicated. The right panel is the band intensity of UCP-1 relative to β-tubulin *(n=4).* **(g)** The mRNA abundance of the thermogenic genes *PGC-1α, Cidea and Dio2* in BAT of the A-FABP KO mice *(n=4).* Uncropped western blot images are shown in Supplementary Fig. 15. Data are represented as mean ± s.e.m. **p<0.05, **p<0.01* (One-way ANOVA with Bonferroni correction for multiple comparisons).

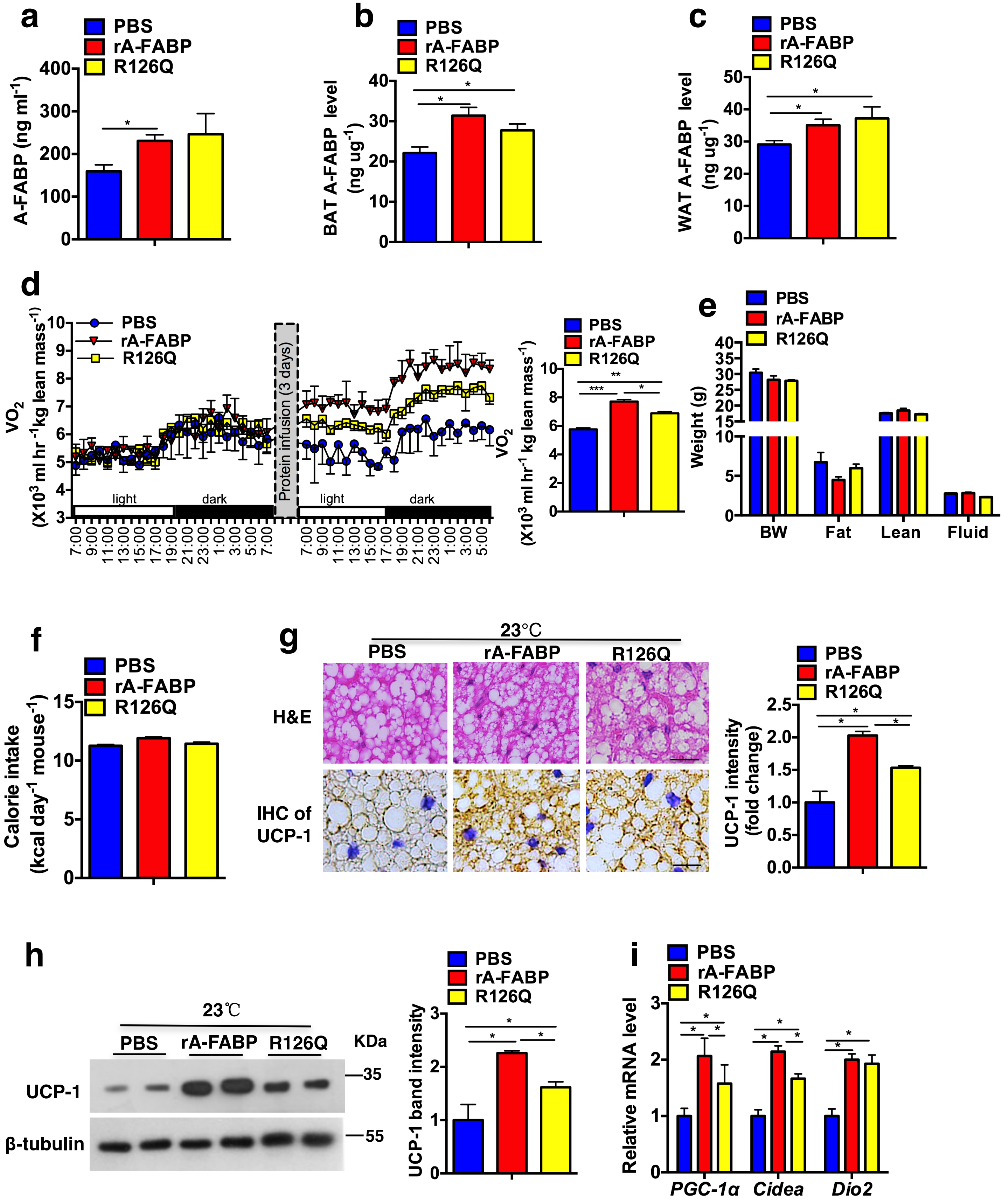
a
PBS
rA-FABP
R126Q
A-FABP (ng ml-1)
b
BAT A-FABP level (ng ug-1)
c
WAT A-FABP level (ng ug-1)
d
VO2 (X10^3 ml hr-1kg lean mass-1)
light
dark
Protein infusion (3 days)
7:00
9:00
11:00
13:00
15:00
17:00
19:00
21:00
23:00
1:00
3:00
5:00
e
Weight (g)
BW
Fat
Lean
Fluid
f
Calorie intake (kcal day-1 mouse-1)
g
23°C
H&E
IHC of UCP-1
UCP-1 intensity (fold change)
h
UCP-1
β-tubulin
KDa
35
55
UCP-1 band intensity
i
Relative mRNA level
PGC-1α
Cidea
Dio2

Supplementary Figure 7. Replenishment of recombinant A-FABP enhances energy expenditure and BAT recruitment in wild-type mice. Male 4-week-old C57BL/6N mice fed with HFD for 4 weeks were infused with PBS, rA-FABP (1 μg $hour^{-1}$) or R126Q (1 μg $hour^{-1}$) and housed at 23 °C for 14 days. **(a)** Circulating A-FABP level, **(b)** A-FABP level in BAT and **(c)** WAT of WT mice after infusion with recombinant proteins *(n=4).* **(d)** Oxygen consumption (VO_2) before or after infusion of PBS, rA-FABP or R126Q. The right panel is the mean VO_2 measured after infusion with recombinant proteins for 3 days *(n=4)*. **(e)** Body weight, body composition and **(f)** calorie intake of the mice. **(g)** Representative H&E staining and IHC staining of UCP-1 in BAT, scale bar=20 μm; with magnification of 400x. Representative images from three independent experiments are shown *(n=4).* **(h)** BAT isolated from above C57BL/6N mice were subjected to immunoblotting using an antibody against UCP-1 and β-tubulin as indicated. The right panel is the band intensity of UCP-1 relative to β-tubulin. **(i)** The mRNA abundance of the thermogenic genes *PGC-1α, Cidea and Dio2* in BAT of the mice *(n=4).* Uncropped western blot images are shown in Supplementary Fig. 15. Data are represented as mean ± s.e.m. *$^*p<0.05$, $^{**}p<0.01$, $^{***}p<0.001$* (One-way ANOVA with Bonferroni correction for multiple comparisons).

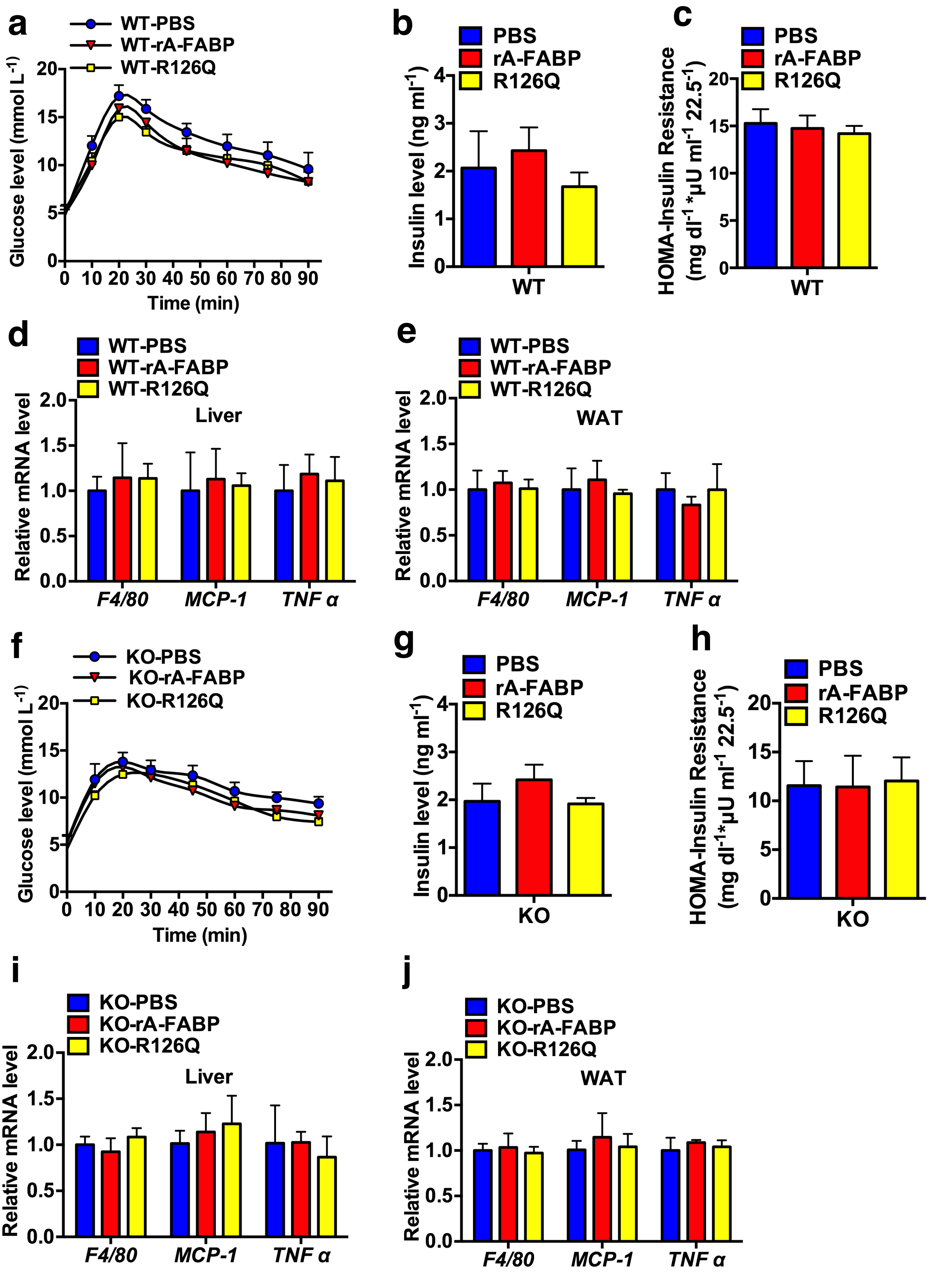
a
WT-PBS
WT-rA-FABP
WT-R126Q
Glucose level (mmol L-1)
Time (min)
b
PBS
rA-FABP
R126Q
Insulin level (ng ml-1)
WT
c
PBS
rA-FABP
R126Q
HOMA-Insulin Resistance (mg dl-1 *μU ml-1 22.5-1)
WT
d
WT-PBS
WT-rA-FABP
WT-R126Q
Liver
Relative mRNA level
F4/80
MCP-1
TNF α
e
WT-PBS
WT-rA-FABP
WT-R126Q
WAT
Relative mRNA level
F4/80
MCP-1
TNF α
f
KO-PBS
KO-rA-FABP
KO-R126Q
Glucose level (mmol L-1)
Time (min)
g
PBS
rA-FABP
R126Q
Insulin level (ng ml-1)
KO
h
PBS
rA-FABP
R126Q
HOMA-Insulin Resistance (mg dl-1*μU ml-1 22.5-1)
KO
i
KO-PBS
KO-rA-FABP
KO-R126Q
Liver
Relative mRNA level
F4/80
MCP-1
TNF α
j
KO-PBS
KO-rA-FABP
KO-R126Q
WAT
Relative mRNA level
F4/80
MCP-1
TNF α

Supplementary Figure 8. Short–term infusion of recombinant A-FABP does not affect glucose metabolism, insulin sensitivity and inflammatory status in A-FABP KO mice and their WT littermates. Male 4-week-old A-FABP KO mice and their WT littermates fed with HFD for 4 weeks were infused with rA-FABP (1µg $hour^{-1}$) or R126Q (1µg $hour^{-1}$) for 14 days. **(a)** Glucose tolerance test (GTT), **(b)** insulin level and **(c)** HOMA index of WT mice. **(d-e)** The mRNA abundance of *F4/80*, *MCP-1* and *TNFα* in (**d**) liver and **(e)** epididymal fat of WT mice. **(f)** Glucose tolerance test (GTT), **(g)** insulin level and **(h)** HOMA index of A-FABP KO mice. **(i-j)** The mRNA abundance of *F4/80*, *MCP-1* and *TNFα* in **(i)** liver and **(j)** epididymal fat of A-FABP KO mice *(n=4).* Data are represented as mean ± s.e.m.

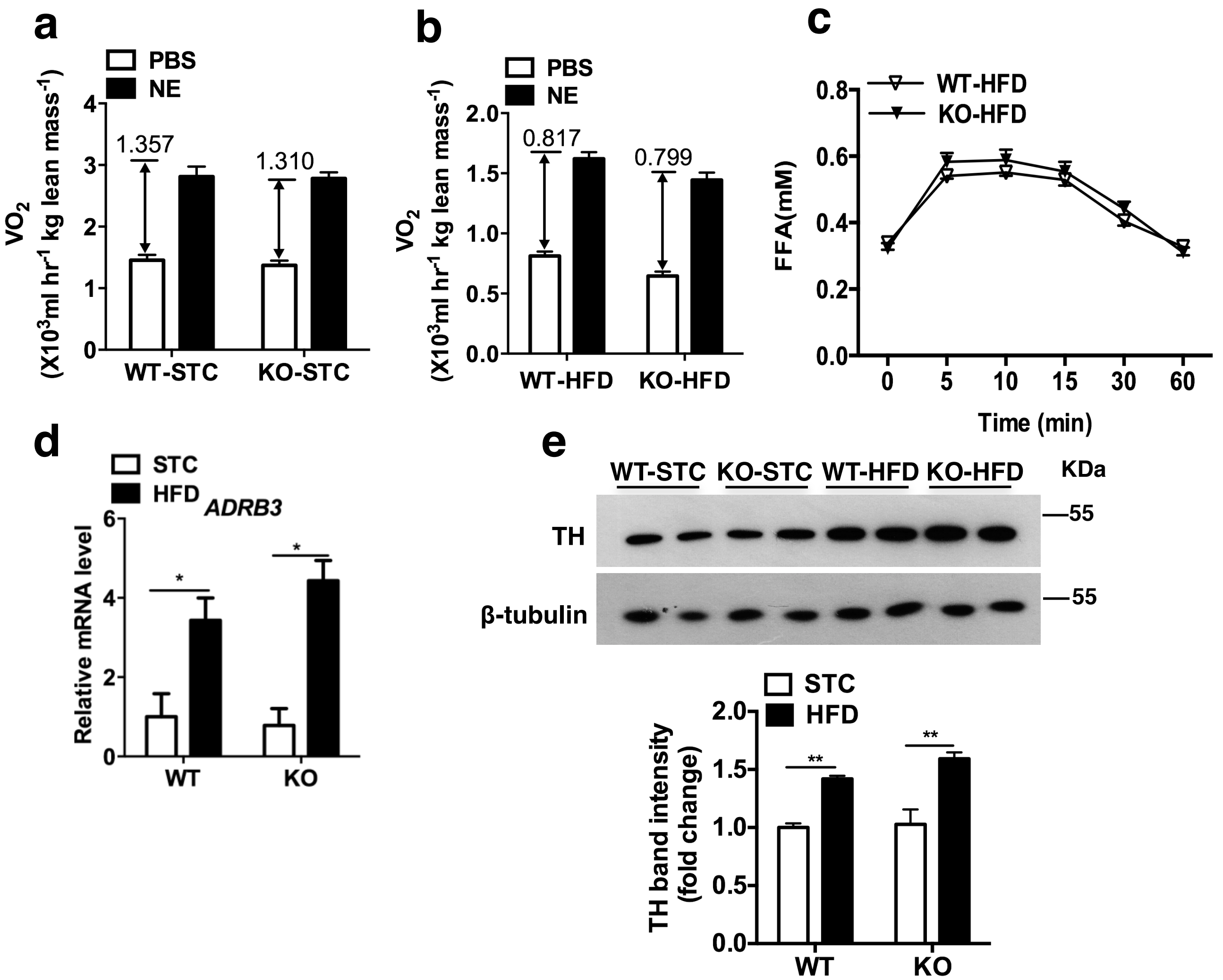

Supplementary Figure 9. A-FABP deficiency does not affect norepinephrine-induced thermogenesis, lipolytic machinery or the activity of sympathetic nervous system in adipose tissue of mice. Male 4-week-old A-FABP KO and their WT littermates were fed with STC or HFD for 24 weeks. **(a-b)** Norepinephrine (NE,1 mg kg^{-1}, i.p)-induced whole body energy expenditure of WT and A-FABP KO mice fed with **(a)** STC or **(b)** HFD *(n=6)*. **(c)** The NE-induced serum FFA level of HFD-induced WT and A-FABP KO mice *(n=6).* **(d)** The mRNA abundance of β adrenergic receptor 3 (*ADRB3*) in BAT of A-FABP KO and WT mice fed with STC or HFD *(n=6).* **(e)** BAT isolated from above A-FABP KO and WT mice fed with STC or HFD were subjected to immunoblotting using an antibody against tyrosine hydroxylase (TH), β-tubulin as indicated. The right panel is the band intensity of TH relative to β-tubulin and expressed as arbitrary units *(n=6).* Uncropped western blot images are shown in Supplementary Fig. 15. Data are represented as mean ± s.e.m. *$*p<0.05$* (Students' t-test).

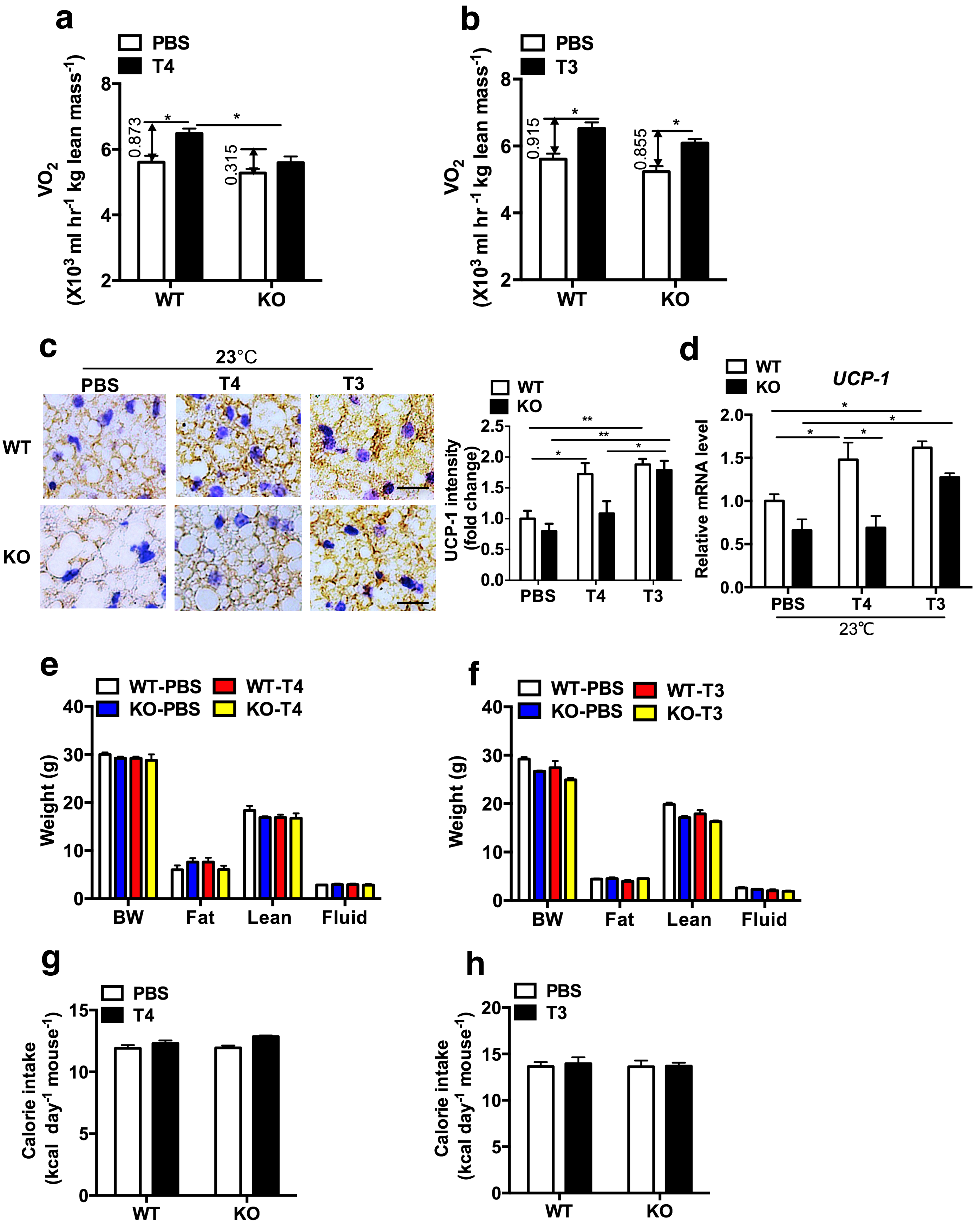
a
PBS
T4
VO2
(X10^3 ml hr^-1 kg lean mass^-1)
8
6
4
2
0.873
0.315
*
*
WT
KO
b
PBS
T3
0.915
0.855
WT
KO
c
23°C
PBS
T4
T3
WT
KO
UCP-1 intensity
(fold change)
2.5
2.0
1.5
1.0
0.5
0.0
**
**
*
*
d
UCP-1
Relative mRNA level
23℃
e
WT-PBS
KO-PBS
WT-T4
KO-T4
Weight (g)
40
30
20
10
0
BW
Fat
Lean
Fluid
f
WT-PBS
KO-PBS
WT-T3
KO-T3
g
PBS
T4
Calorie intake
(kcal day^-1 mouse^-1)
15
10
5
0
h
PBS
T3
20

Supplementary Figure 10. Energy expenditure, BAT recruitment and basic parameters of WT and A-FABP KO mice treated with T4 or T3 under room temperature (23 °C). Male 4-week-old A-FABP KO and their WT littermates fed with HFD for 4 weeks were housed at 23 °C and subcutaneously injected with T4 (400 μg kg^{-1}, 5 days) or T3 (500 μg kg^{-1}, 1 day). **(a-b)** Energy expenditure (normalized with lean mass) of mice treated with **(a)** T4 or **(b)** T3 *(n=4)*. **(c)** Representative IHC staining and densitometry analysis (right panel) for UCP-1 in BAT of the mice treated with T4 or T3. Scale bar=20 μM, with magnification of 400x. Representative images from three independent experiments are shown *(n=4)*. **(d)** The mRNA abundance of UCP-1 in BAT of the mice. **(e-f)** Body weight and body composition of the mice treated with **(e)** T4 or **(f)** T3. **(g-h)** Calorie intake of the mice treated with **(g)** T4 or **(h)** T3 *(n=4)*. Data are represented as mean ± s.e.m. *p<0.05, **p<0.01 (One-way ANOVA with Bonferroni correction for multiple comparisons).

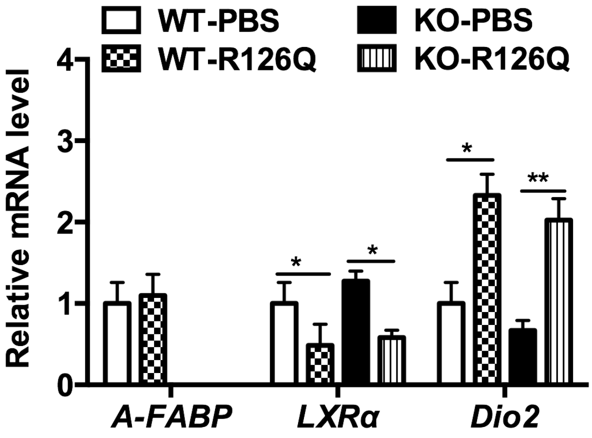

Supplementary Figure 11. The A-FABP mutant R126Q induces expression of *Dio2* via inhibition of LXRα. The mRNA abundance of *A-FABP*, *LXRα* and *Dio2* in WT or A-FABP deficient primary brown adipocytes derived from male 6-week-old WT and A-FABP KO mice incubated with A-FABP mutant R126Q (2 μg ml^{-1}) or PBS for 24 hours was determined by real-time PCR *(n=6).* Data are represented as mean ± s.e.m. *$*p<0.05$, $**p<0.01$* (One-way ANOVA with Bonferroni correction for multiple comparisons).

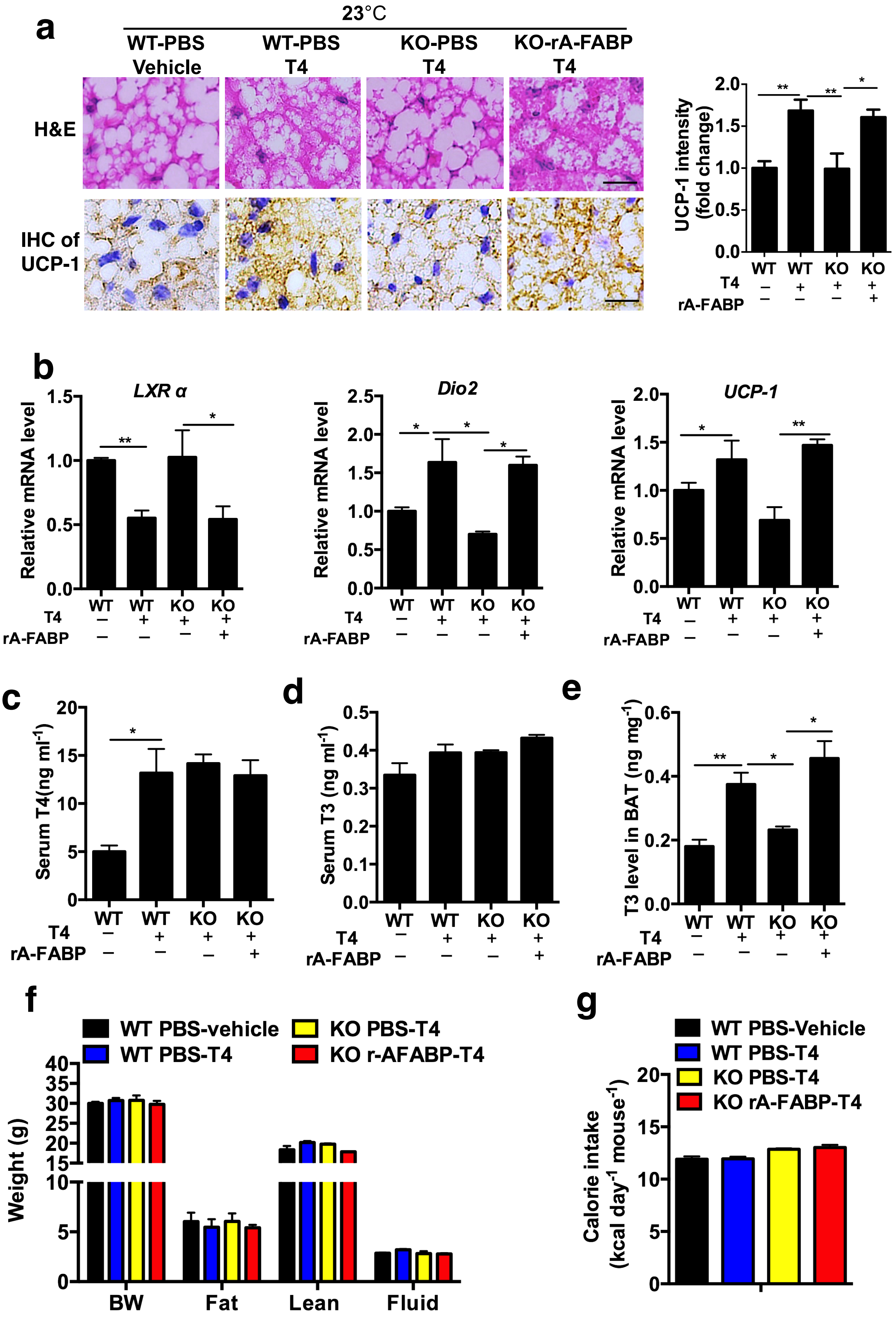
a
23°C
WT-PBS Vehicle
WT-PBS T4
KO-PBS T4
KO-rA-FABP T4
H&E
IHC of UCP-1
UCP-1 intensity (fold change)
T4
rA-FABP
b
LXR α
Dio2
UCP-1
Relative mRNA level
c
Serum T4(ng ml-1)
d
Serum T3 (ng ml-1)
e
T3 level in BAT (ng mg-1)
f
WT PBS-vehicle
WT PBS-T4
KO PBS-T4
KO r-AFABP-T4
Weight (g)
BW
Fat
Lean
Fluid
g
WT PBS-Vehicle
WT PBS-T4
KO PBS-T4
KO rA-FABP-T4
Calorie intake (kcal day-1 mouse-1)

Supplementary Figure 12. A-FABP enhances conversion of thyroid hormones in BAT of A-FABP KO mice at 23 °C. Male 4-week-old A-FABP KO mice and their WT littermates fed with HFD for 4 weeks were housed at 23 °C and infused with rA-FABP (1μg hour^{-1},14 days) or PBS followed by subcutaneous injection of T4 (400 μg kg^{-1}; 5 days). **(a)** Representative H&E staining and IHC staining of UCP-1 and densitometry analysis for the expression of UCP-1 (right panel) in BAT. Scale bar=20 μm; with magnification of 400x. Representative images from three independent experiments are shown *(n=4).* **(b)** The mRNA abundance of *LXRα, Dio2 and UCP-1* in BAT of the mice. Circulating levels of **(c)** T4, **(d)** T3 and **(e)** T3 level in BAT of the mice *(n=4).* **(f)** Body weight, body composition and **(g)** calorie intake of the mice (*n*=4)**.** Data are represented as mean ± s.e.m. $*p<0.05$, $**p<0.01$ (One-way ANOVA with Bonferroni correction for multiple comparisons).

Fig 2d

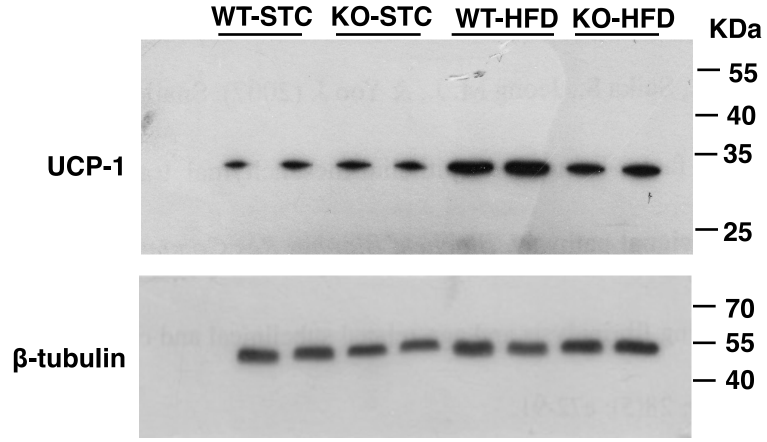

Fig 2e

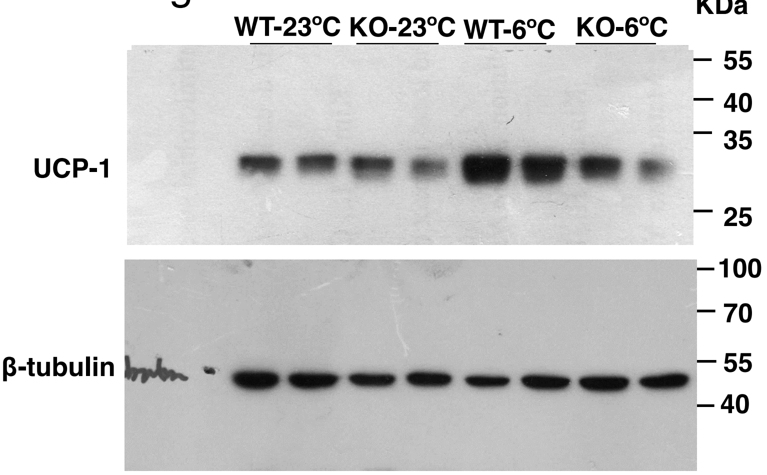

Fig 3g

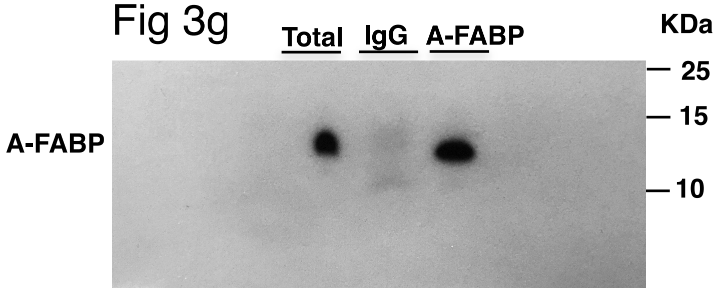

Fig 4f

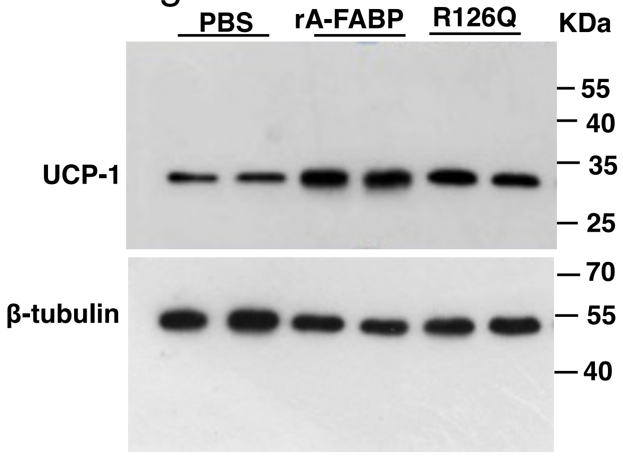

Fig 6a

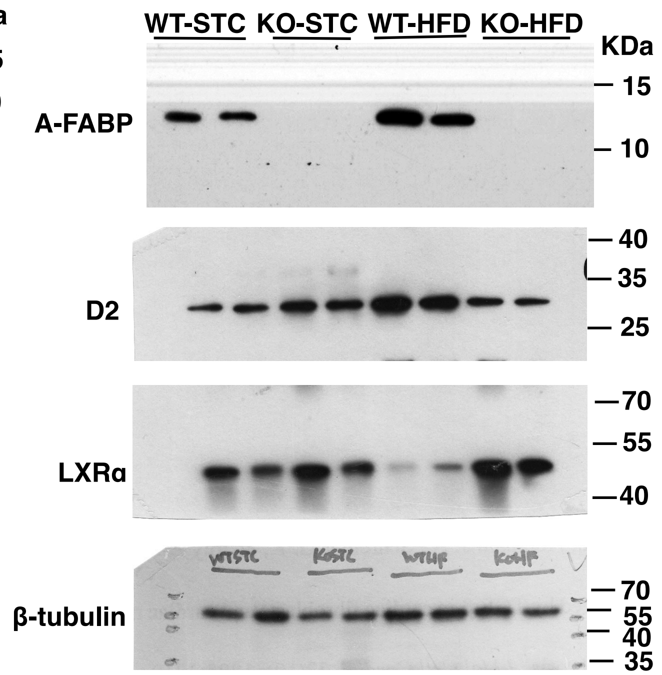

Fig 6b

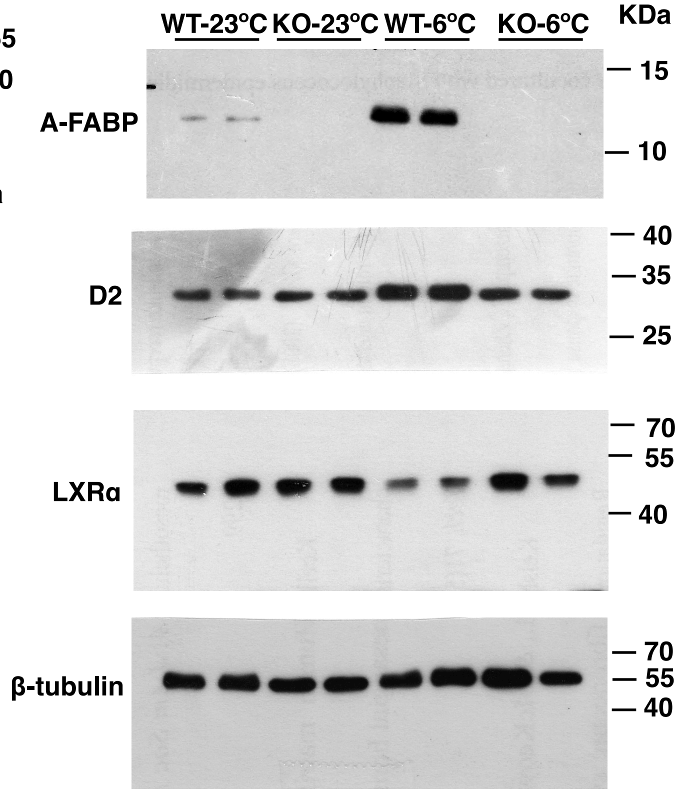

Supplementary Figure 13. Uncropped scans of the immunoblots (Figure 2,3,4,6).

Fig 7b

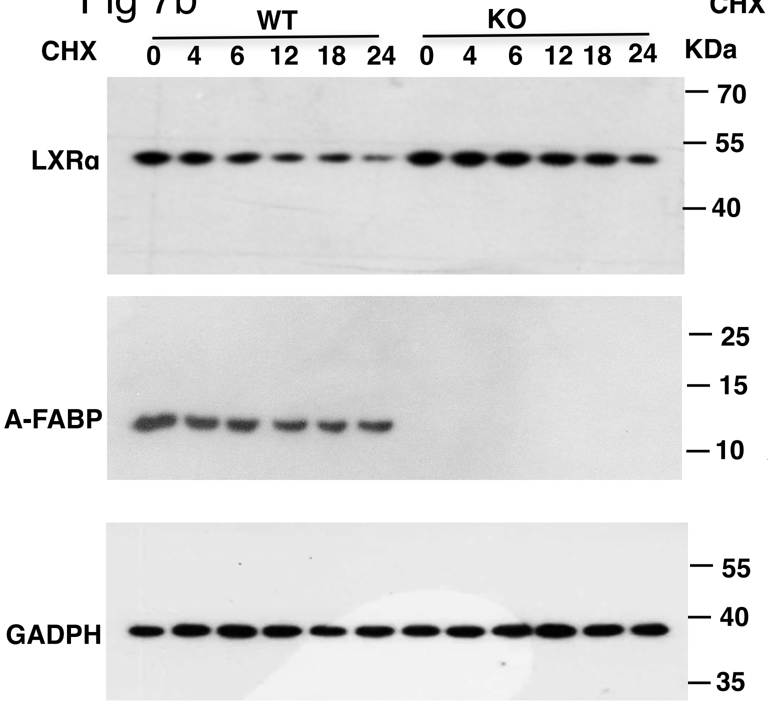

Fig 7d

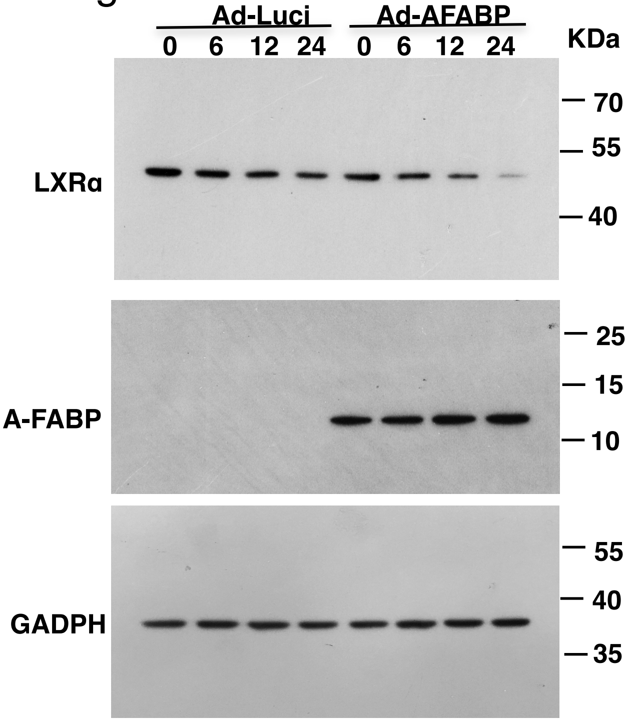

Fig 7c

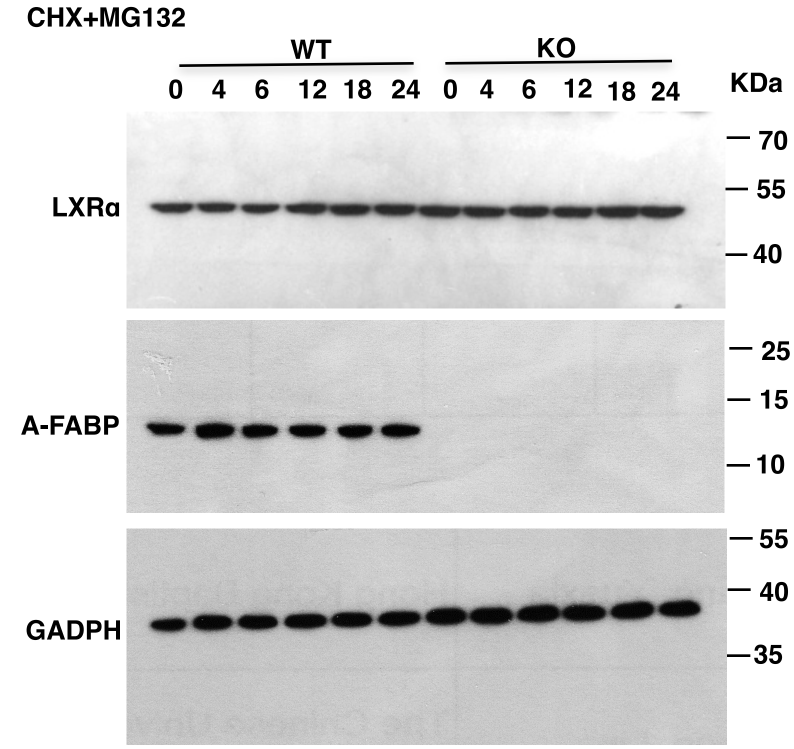

Supplementary Figure 14. Uncropped scans of the immunoblots (Figure 7).

Supplementary Fig 4a

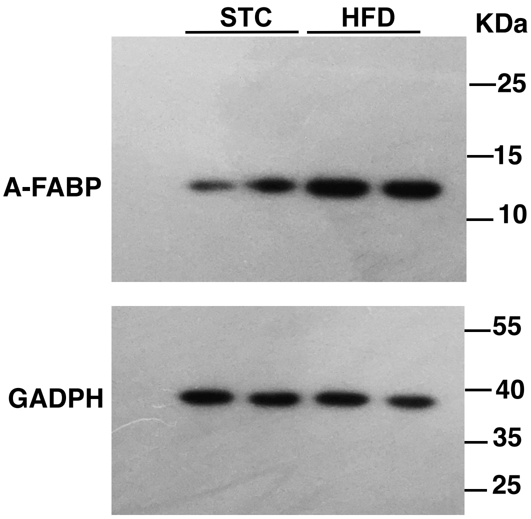

Supplementary Fig 7h

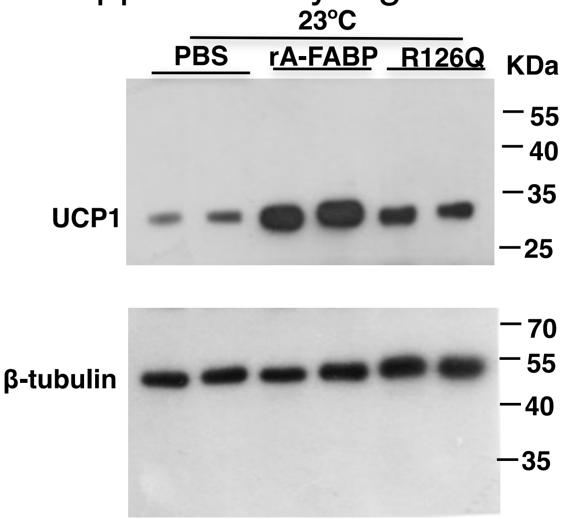

Supplementary Fig 4b

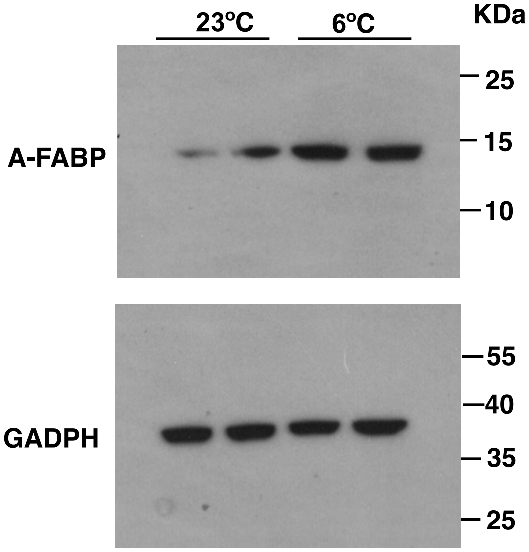

Supplementary Fig 9e

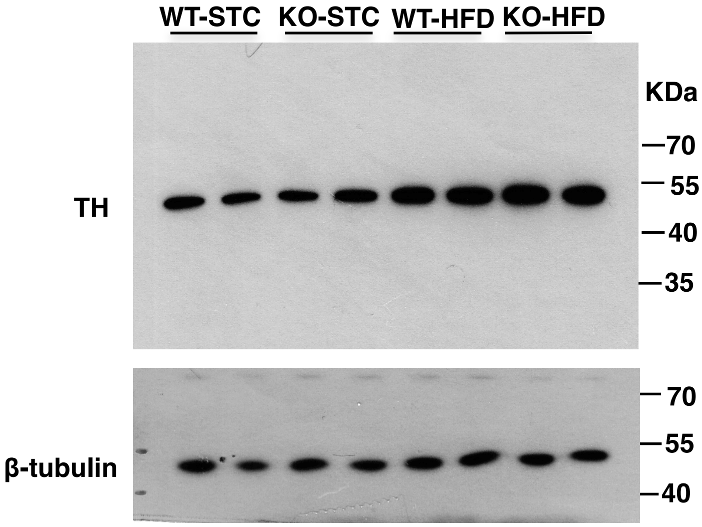

Supplementary Fig 6f

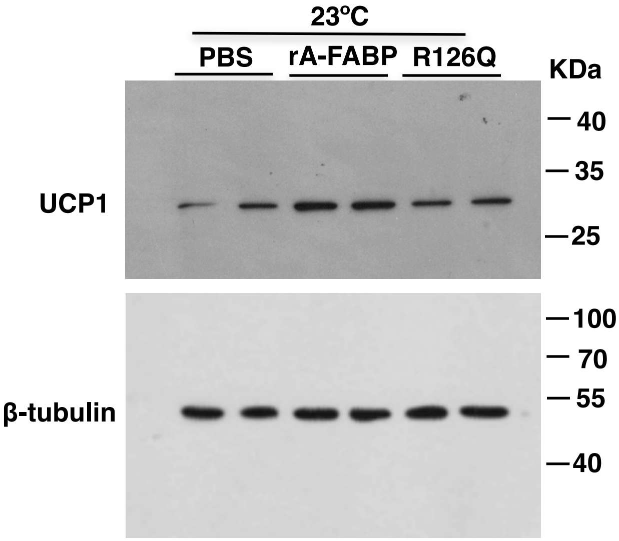

Supplementary Figure 15. Uncropped scans of the immunoblots (Supplementary Fig 4,6,7, 9).

1. Sequences of primers used for site-directed mutagenesis for generation of the A-FABP mutant R126Q

Name	Forward	Reverse
R126Q	GGCGTGACTTCCACACACAAGTTTAT GAAAGGGCATG	CATGCCCTTTCATAAACTTGTGTGGAAGTCA CGCC

2. Sequences of primers used for real-time PCR analysis

Name	Forward	Reverse
A-FABP	ACA CCG AGA TTT CCT TCA AAC TG	CCA TCT AGG GTT ATG ATG CTC TTC A
PGC-1α	CCC TGC CAT TGT TAA GAC C	TGC TGC TGT TCC TGT TTT C
Cidea	TGC TCT TCT GTA TCG CCC AGT	GCC GTG TTA AGG AAT CTG CTG
Dio2	CAG TGT GGT GCA CGT CTC CAA TC	TGA ACC AAA GTT GAC CAC CAG
LXRα	CCT TCC TCA AGG ACT TCA GTT ACA A	CAT GGC TCT GGA GAA CTC AAA GAT
SCD-1	CAT CAT TCT CAT GGT CCT GCT	CCC AGT CGT ACA CGT CAT TTT
SREBP-1c	GTT ACT CGA GCC TGC CTT CAG G	CAA GCT TTG GAC CTG GGT GTG
ADRB3	TCCGTTTTTGCAGGACTTCT	ACGGTGAAACCCATTTGGTA
CD 36	GGC ACC ACT GTG TAC AGA CAG	GGA AAG GAG GCT GCG TCT GTG C
FATP-1	TCACTGGCGCTGCTTTGGTT	GGACGTGGCTGTGTATGG

Supplementary Table 1. Sequences of primers used in this study.
